# Analysis of Research Trends in Ultrasound-Guided Acupuncture and Dry-Needling: A Scoping Review

**DOI:** 10.3390/jcm13164962

**Published:** 2024-08-22

**Authors:** Hyunwook Shin, Hyeonjun Woo, Yunhee Han, Seungkwan Choi, Jungho Jo, Seojae Jeon, Wonbae Ha, Junghan Lee

**Affiliations:** 1 Department of Korean Medicine, College of Korean Medicine, Wonkwang University, Iksan 54538, Republic of Korea; alex0513@naver.com; 2 Department of Korean Medicine Rehabilitation, College of Korean Medicine, Semyung University, Jecheon 27136, Republic of Korea; woohyeonjun@gmail.com; 3 Department of Korean Medicine Rehabilitation, College of Korean Medicine, Wonkwang University, Iksan 54538, Republic of Korea; kmedhyh@gmail.com (Y.H.); sskkchoi12@gmail.com (S.C.); 4 Department of Korean Internal Medicine, College of Korean Medicine, Wonkwang University, Iksan 54538, Republic of Korea; jjh4972@naver.com; 5 Korea Institute of Integrative Medicine, Jangheung 59301, Republic of Korea; fr1771@naver.com; 6 Research Center of Traditional Korean Medicine, College of Korean Medicine, Wonkwang University, Iksan 54538, Republic of Korea

**Keywords:** acupuncture, dry-needling, needle knife, acupotomy, pharmacopuncture, ultrasound, ultrasound-guided

## Abstract

**Background:** This study aimed to summarize the current status of research on ultrasound-guided acupuncture and dry-needling treatment and the specific treatment methods applied to patients. **Methods:** A scoping review was conducted, surveying three English databases (PubMed, Embase, and the Cochrane Library) for studies published up to May 2024. All studies related to ultrasound-guided acupuncture and dry-needling treatment were considered. Literature was selected using selection and exclusion criteria, and extracted and organized using EndNote. **Results:** A total of 107 eligible studies were included. Among the 107 studies, non-comparative studies accounted for the largest proportion (n = 47, 43.9%), followed by randomized controlled trials (RCTs; n = 41, 38.3%). Diseases of the musculoskeletal system or connective tissue (15 diseases of the musculoskeletal system or connective tissue) accounted for most (n = 48, 55.8%) of the 86 diseases studied, followed by symptoms, signs, or clinical conditions not otherwise classified (n = 17, 19.8%). **Conclusions:** Ultrasound-guided acupuncture and dry-needling have been actively studied and applied for the treatment of various diseases. However, higher-quality studies are needed for further applications in research and clinical practice.

## 1. Introduction

Acupuncture originated in Chinese medicine; traditionally, its main purpose is to stimulate specific points called “acupuncture points” along meridians to restore qi flow and activate metabolism. Today, the term has been expanded to include dry-needling, which is applied locally to areas where trigger points (TrPs) exist and myofascial pain. Referred pain, also known as secondary hyperalgesia, is commonly associated with TrPs and occurs when pain is experienced in a different region than its source, a phenomenon frequently observed in nearly all myofascial pain syndromes [1]. At medical institutions, patient complaints are followed by a series of tests, and clinician experience is used to diagnose and treat the disease.

However, the commonly used treatment methods of acupuncture and dry-needling are invasive, and the needling process is largely dependent on the practitioner’s senses. Variations in age, sex, body type, and anatomy exist among patients; thus the stability of needle depth and direction and the reproducibility of treatment according to the skill of the practitioner must be studied [2]. Ultrasound imaging (ultrasonography) utilizes unique acoustic impedance differences between the internal tissues of the body to transmit a constant pulse wave into the body, and the reflected signal is amplified and converted into a computerized image [3]. Ultrasound devices are used as diagnostic and therapeutic aids to improve the accuracy and precision of treatment for non-palpable areas because they can help identify the location of lesions and morbidity [4,5]. Therefore, research has been conducted on techniques that combine ultrasound-guided acupuncture and dry-needling to improve the accuracy and precision of existing acupuncture and dry-needling techniques.

Until now, most studies combining acupuncture and dry-needle therapy with ultrasound imaging devices have focused on the treatment of a single disease in patients. Therefore, studies are lacking regarding the overall research status and possibilities of ultrasound-guided acupuncture and dry-needle therapy for researchers and clinical practitioners.

Therefore, in this scoping review, we systematically searched for and analyzed studies related to ultrasound-guided acupuncture (acupuncture, acupotomy, and pharmacopuncture) and dry-needling applications to investigate the current state of research on ultrasound-guided acupuncture and dry-needling. We aimed to identify specific methods of ultrasound-guided acupuncture and dry-needling according to the disease and site, and explore the possibility of further applications to provide reference materials for clinicians.

## 2. Materials and Methods

A scoping review was conducted to identify research trends in ultrasound-guided acupuncture and dry-needling. By broadening the scope of the question, the following research questions were set to identify areas where clinical research on ultrasound-guided acupuncture and dry-needling is insufficient.

1. What are the research trends in ultrasound-guided acupuncture and dry-needling? (Publication year, research design, etc.)
2. For what specific diseases or symptoms are ultrasound-guided acupuncture and dry-needling used?
3. What are the future research directions for ultrasound-guided acupuncture and dry-needling?

Based on Arksey and O’Malley’s five steps [6], the authors drafted this study under the guidance of the Preferred Reporting Items for Systematic Reviews and Meta-Analyses (PRISMA) [7] extension of the scoping review checklist and Supplementary Materials.

### 2.1. Database Selection and Search

A literature search was conducted using the PubMed (https://pubmed.ncbi.nlm.nih.gov), EMBASE (www.embase.com), and Cochrane Library (www.cochranelibrary.com) databases. Until 30 May 2024, we searched for articles reported, using terms such as “ultrasound”, “ultrasound-guided”, “sonography”, “ultrasonogram”, “ultrasonogram”, and “echography” for ultrasound-related terms and “acupuncture” and “acupuncture therapy” for acupuncture and dry-needling-related terms. Term such as “shonishin”, “needling”, “electroacupuncture”, “acupotomy”, “acupotome”, “dry-needling”, “needle knife”, “miniscalpel needle” [8], “pharmacopuncture”, and “herb acupuncture” were combined to search for articles containing these words in the title/abstracts. Two researchers (SHW, HWB) independently searched databases. The full search strategy for the database is provided in Appendix S1.

### 2.2. Inclusion and Exclusion Criteria

We included studies that met the following criteria

1. Journal articles related to ultrasound-guided acupuncture among the retrieved studies
2. Articles that included ultrasound and acupuncture-related keywords in the title or abstract
3. Systematic reviews, meta-analyses, randomized controlled trials (RCT), case reports, case series, non-RCTs, non-comparative studies, reviews, cohort studies, and cross-sectional studies

Studies that met the following criteria were excluded

1. Studies that used ultrasound only in the diagnostic process of a disease
2. Studies that did not involve human subjects: methodological studies, literature reviews, etc. (except systematic reviews of clinical studies)
3. Studies whose full text could not be verified
4. Conference presentations, research protocols, commentaries, letters, and editorials

### 2.3. Study Selection and Data Extraction Analysis

The bibliographic program Endnote x9 (Clarvate Analytics, Philadelphia, PA, USA) was used to search and organize the data, while Microsoft Excel 2016 (Microsoft, Redmond, WA, USA) was used to record the data. Study selection was based on the inclusion and exclusion criteria established in a meeting of the researchers. Titles were reviewed to exclude irrelevant studies, and abstracts were reviewed for the final selection. The search was not restricted by the country in which the study was conducted or by the age, sex, or language of the participants. Two researchers independently reviewed the literature; if any disagreement arose regarding the process and results of evaluating the literature, a third researcher was consulted to reconcile their opinions.

The researchers determined the data items to be extracted as the main items related to the initial research questions, such as year of publication, author, study type, intervention method, target disease, number of experimental/control groups, treatment site, evaluation indicators, and outcomes. Two researchers independently conducted data extraction, and finally, a third researcher reviewed the data and recorded the agreement after exchanging opinions and discussions.

### 2.4. Data Analysis, Summarization, and Reporting of Results

The overall trends of analytics school research in terms of publication year, academic discipline, and research design were recorded. This study aimed to provide the main information and results related to this research topic, wherein the scope of application of the concept of ultrasound-guided acupuncture and dry-needling treatment was analyzed based on the process of the intervention and the clinical symptoms of the subjects. The main findings are described using PRISMA diagrams and are presented in the tables and figures [9].

## 3. Results

### 3.1. Article Search and Selection Results

**Figure 1:**
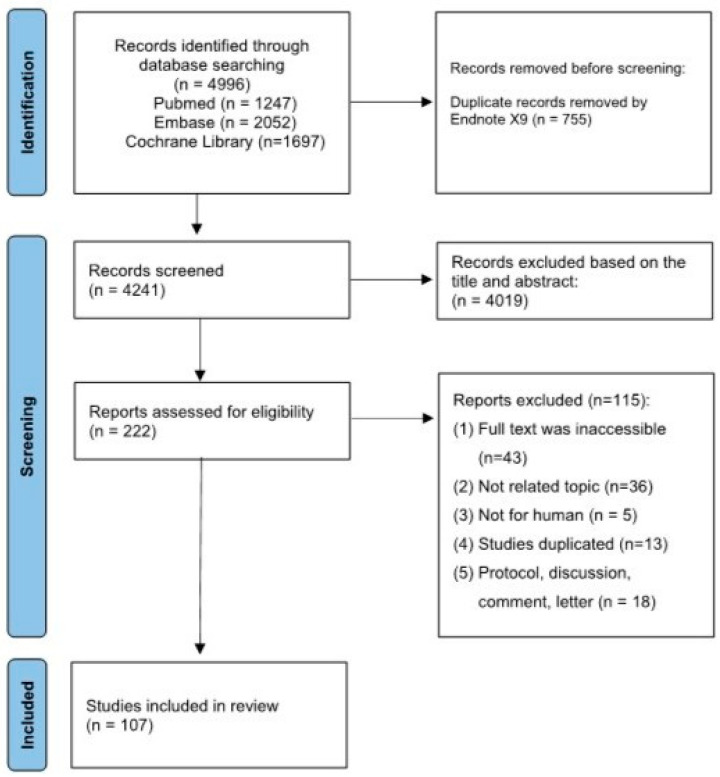
Flowchart of the study selection process.

Of the 4996 articles retrieved from the database, 107 were selected for scoping review (Figure 1). 

### 3.2. Research Trends

#### 3.2.1. Year of Publication

**Figure 2:**
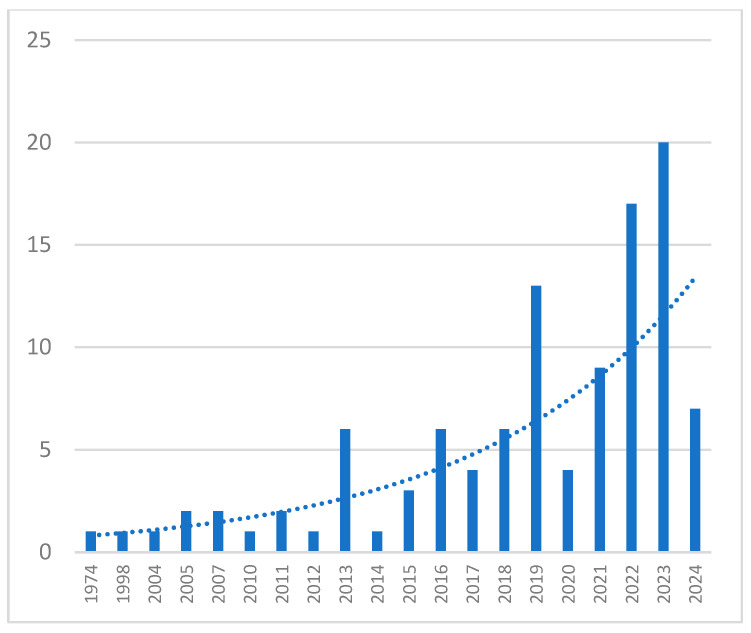
The number of studies published in each year.

To identify trends in the volume and content of research over time, the years of publication of ultrasound-guided acupuncture-related studies were analyzed. Since 1974, 2023 was the most active year, with 20 studies (18.7%), followed by 2022, with 17 studies (15.9%) (Figure 2).

#### 3.2.2. Study Design

The study design was based on the clinical research literature classification tool DAMI (version 2.0; Health Insurance Review and Assessment Institute, Seoul, Korea) [10], which involved reviewing the abstracts and full texts. Of the 107 studies, non-comparative studies accounted for the largest proportion (n = 47, 43.9%), followed by randomized controlled trials (RCTs), (n = 41, 38.9%). This was followed by four cross-sectional studies and reviews (3.7%), three meta-analyses and non-randomized controlled trials (nRCTs) (2.8%), two prospective cohort studies (1.9%), and one systematic review, pilot study, and reliability study each (0.9%) (Table 1).

#### 3.2.3. Disease Analysis

The diseases studied were categorized according to the ICD-11 criteria. Four reviews of clinical studies [11,12,13,14], one systematic review [15], and three meta-analyses [16,17,18] were excluded because of possible duplications in the analysis of target diseases. Furthermore, 28 studies that were not disease specific, such as those on the safety of needle depth, anatomical approaches, and needle pain or sensation, were excluded. A total of 71 papers were analyzed, and when more than one disease classification was present within a study, each disease or symptom was counted separately. Of the 86 disease categories, category 15, “Diseases of the musculoskeletal system or connective tissue” was reported in 48 (55.8%) cases, followed by category 21, “Symptoms, signs, or clinical findings not classified elsewhere” being observed in 17 (19.8%) cases. Among diseases of the musculoskeletal system or connective tissue, FA01, “Osteoarthritis of knee”, was the most common, with 6 (7.0%) cases For 21, (“Symptoms, signs, or clinical findings not otherwise specified”) MG30.01, “Chronic widespread pain”, was the most common with 10 (11.6%) cases, followed by MG30.02, “Chronic primary musculoskeletal pain” with 3 (3.5%) cases (Appendix S3).

### 3.3. Analysis by Study Type

#### 3.3.1. RCTs and Non-RCTs

A total of forty-one RCTs and three non-RCTs were retrieved, with the following results. For RCTs, the most common study design (13 studies) [19,20,21,22,23,24,25,26,27,28,29,30,31] involved the experimental group receiving ultrasound-guided acupuncture or dry-needling and the control group receiving treatments other than acupuncture or dry-needling (e.g., surgery [19], high-energy extracorporeal shock wave [20,32]), followed by studies (11 studies [27,33,34,35,36,37,38,39,40,41,42]) that compared the effects of ultrasound-guided acupuncture or dry-needling in the experimental group and the same treatment but without ultrasound guidance in the control group. Nine studies [32,41,43,44,45,46,47,48] used the same treatment in both the experimental and control groups, with the addition of ultrasound-guided acupuncture or dry-needling in the experimental group only, while nine studies [49,50,51,52,53,54,55,56,57] used ultrasound-guided acupuncture or dry-needling in both the experimental and control groups, but with subtle differences. One non-RCT compared an experimental group that received localized intense stimulation electroacupuncture using B-ultrasonography with two control groups that received medication and conventional acupuncture, respectively [58]. Other non-RCTs compared the result of ultrasound-guided needling in the experimental group and the same intervention but without ultrasound guidance in the control group [59,60].

**Figure 3:**
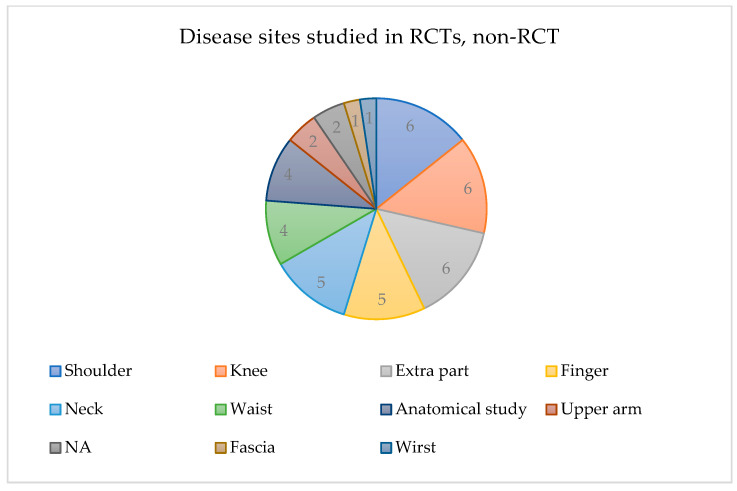
Disease sites studied in RCTs and non-RCT.

The specific interventions applied in the RCTs were mainly ultrasound-guided acupuncture and dry-needling. Involved in ultrasound-guided dry-needling studies were lidocaine [33,34], intrathecal injections [43], extracorporeal shock waves [44], juanbi-decoction [51], steroid injections [47], drug injections [61], and hydrodilatation [48], dry-needling combined with extracorporeal shock wave treatment [20,32], percutaneous electrotherapy [22], physical therapy [45,46], and the Mulligan technique [25]. Studies also exist regarding electroacupuncture [30] and embedding acupuncture [49,50]. Studies have also compared ultrasound-guided intervention with conventional treatments, such as one comparing ultrasound-guided acupotomy with unguided electroacupuncture (EA) [21]. The shoulder, knee were the most common morbidities studied in RCTs (six each), followed by the neck, finger. (five). The lower back, fasciae, upper arms, wrists, and other anatomical structures were also examined (Figure 3). The evaluation of treatment outcomes was mainly based on the Visual Analog Scale (VAS) and Numeric Rating Scale (NRS). Patients’ VAS and NRS scores decreased before and after ultrasound-guided acupuncture or dry-needling treatment, and functional aspects assessed using the Shoulder Pain and Disability Index (SPADI) [44], Knee Injury and Osteoarthritis Outcome Score (KOOS) [29,45,46], Neck Disability Index (NDI) [50,57], and American Orthopedic Foot and Ankle Society Ankle–Hindfoot scoring system (AOFAS) [34] also improved before and after treatment (Table 2).

#### 3.3.2. Systematic Review and Meta-Analysis

One systematic review was retrieved which included a total of 12 RCTs and 481 participants [15]. The experimental group received percutaneous ultrasound-guided tendonectomy, and the control group received an alternative treatment (surgical tendonectomy, platelet-rich plasma injection, steroid injection). Percutaneous ultrasound-guided needle tenotomy was effective in maintaining improvement after treatment of chronic tendinopathy compared to other alternative treatments.

A total of 3 meta-analyses were retrieved, which included a total of 3205 participants [16,17,18]. In study of ultrasound-guided acupotomy for osteoarthritis, the experimental group that received ultrasound-guided acupotomy showed a reduction in VAS, improvement in knee function on the Lysholm knee score, and a relatively better clinical effectiveness rate compared to the control group. Ultrasound-guided acupotomy was also associated with a lower incidence of adverse events compared to conventional acupuncture (odds ratio = 0.27). In study of ultrasound-guided acupotomy for trigger finger, overall clinical effectiveness (OR = 4.83; 95% CI 2.49–9.37; I2 = 73.1%; *p* < 0.001) in the experimental group was significantly better than that of the control group (Appendix S4).

#### 3.3.3. Non-Comparative Study

A total of 47 non-comparative studies were retrieved, including 14 case reports of ultrasound-guided acupuncture and dry-needling treatments in one patient and 9 case series with multiple patients. Further, 24 clinical trials existed that were not studies on patient treatment with ultrasound-guided acupuncture or dry-needling but on pain sensation or needle sensation, anatomical studies, and others.

##### Case Reports and Case Series 

In total, 14 case reports and 9 case series were retrieved. The ultrasound-guided intervention methods studied were dry-needling in 17 cases [63,64,65,66,67,68,69,70,71,72,73,74,75,76,77,78,79], electroacupuncture in 4 cases [80,81,82,83], and acupotomy in 2 case [84,85]. Dry-needling in patients has been studied in isolation [63,64,65,67,68,69,70,71,72,73,74,76,77,78,79,83], in combination with therapeutic exercise [66], and as an intervention after neurointervention [75]. For electroacupuncture, there have been studies of ultrasound-guided electroacupuncture alone [81,82,83] and in combination with training [80], and for acupotomy, there have been studied in isolation [85], in combination with perineural injections [84]. The diseases studied were mainly pain-related [17,63,65,66,68,69,70,71,72,73,74,75,76,78,79,80,83,84]. The indicators studied in each article were Range of Movement (ROM) [64,66,68,69,81], VAS [63,65,70,71,79,83,84], Symptoms [66,67,72,74,80,82,85], NRS [68,73,75,81], or Symptom Score [69,75,77,78,80].All studies showed some level of effectiveness, with no adverse effects reported (Table 3).

##### Clinical Trial

A total of 24 clinical trials were reviewed. Studies have been conducted on various topics involving ultrasound-guided acupuncture and dry-needling. Differences in patient-perceived sensations depending on needle depth have also been evaluated [86,87]. Studies have also assessed the anatomical characteristics of the treatment area [88,89,90,91,92], changes that occur inside the body after acupuncture treatment [93,94], needle displacement in typical acupuncture cases [95], the usefulness of ultrasound application in acupuncture treatment [96,97], the methodological aspects of the treatment technique [98], the efficacy of ultrasound-guided dry-needling [99,100,101,102], the safety and efficacy of existing dry-needling treatment procedures [103,104,105,106], disease mechanisms [107], and new treatment techniques [108]. Studies using cadavers have also been found [98,102,105,106,109] (Appendix S5).

#### 3.3.4. Prospective Cohort Study

Two cohort studies were included in the scoping review (Appendix S6). In a study of ultrasound-guided tendon needling combined with autologous blood injections in 47 cases of patellar tendinopathy in 44 patients [110], patients’ Victoria Institute of Sport Assessment scores (VISA) improved significantly when pre- and post-treatment outcomes were followed up for a mean of 14.8 months. In a study of ultrasound-guided dry-needling and percutaneous high-dose stripping for Achilles tendinopathy in 64 patients, significant improvements in pain scales and high patient satisfaction were observed [111].

## 4. Discussion

Acupuncture is a therapeutic technique with proven applicability and effectiveness in various diseases, and several studies have been conducted worldwide. Currently, acupuncture is practiced in various ways—including needle knife, pharmacopuncture, and embedding needles—depending on the patient’s symptoms and location. However, given its invasive nature, the reproducibility and safety of the treatment has been questioned since the basic process relies on the practitioner’s facilitation. Although most acupuncture treatments are highly effective and have minimal or no side effects, differences exist in the amount of stimulation to the patient depending on the depth of needling [112,113]. Setting and entering the correct needle point without displacing the needle is essential for quality treatment. As such, studies combining needling techniques with various imaging devices have been actively conducted in foreign countries. However, no study has systematically summarized these techniques or presented them specifically to researchers and clinical practitioners. Therefore, the present study conducted a scoping review to analyze the research trends in ultrasound-guided acupuncture treatment, main applied diseases, specific application methods, and research results, and to suggest future research directions and applications in medical institutions.

Three databases were searched for studies on ultrasound-guided acupuncture and dry-needling. For ultrasound-related terms, we used “ultrasound” and “ultrasound-guided.” For acupuncture and dry-needling-related terms, we used a combination of “acupuncture”, “acupotomy”, “dry-needling”, “needle knife”, “miniscalpel needle [8]”, and “pharmacopuncture” to identify the research status of acupuncture treatments in as many fields as possible. Of the 4996 articles retrieved from the database, 107 were selected for scoping review. Studies have been published every year since 1974, and as of May, 7 studies have already been published in 2024, indicating that research remains active. The study design was categorized based on the DAMI version 2.0, which was developed by the Health Insurance Portability and Accountability Institute in 2013. Of the 107 studies, 47 were categorized as non-comparative studies (43.9%), including 24 clinical trials, 14 single-case reports, and 9 case series. RCTs accounted for 41 studies (38.3%). The high proportion of non-comparative studies and RCTs may be because ultrasound-guided acupuncture and dry-needling are new treatment modalities that have not been widely used; therefore, further research on this technique is needed. Six articles that did not specify the sample size of the clinical study were excluded.

In terms of disease classification, 48 studies (55.8%) focused on diseases of the musculoskeletal system or connective tissues. In terms of site classification, most studies focused on joints such as the shoulders and knees. This may be due not only to the fact that acupuncture and dry-needling are gaining attention as nonpharmacologic treatments for pain management, especially in the musculoskeletal system, but also because their effectiveness has been demonstrated, leading to further research in this area. In addition, studies have been conducted on patients treated with ultrasound-guided acupuncture and dry-needling for a variety of conditions, including certain infectious or parasitic diseases, neoplasms, endocrine disorders, nutritional metabolic disorders, neurological disorders, respiratory disorders, genitourinary disorders, and the consequences of trauma or poisoning, with excellent results. These findings suggest that ultrasound-guided acupuncture and dry-needling are not merely limited to musculoskeletal disorders and pain, but can also be applied to a wide range of other conditions.

As for RCTs, 13 studies were conducted wherein the experimental group was treated with ultrasound-guided acupuncture and dry-needling and the control group was treated with modalities other than acupuncture and dry-needling. The next most common experimental and control group design was found in 11 studies, in which the experimental group was treated with ultrasound-guided acupuncture and dry-needling, and the control group was treated with the same acupuncture and dry-needling modalities but without ultrasound guidance. These research trends are primarily driven by studies aimed at confirming the inherent safety of ultrasound-guided acupuncture and dry-needling techniques, as well as exploring the efficacy of adding ultrasound guidance to existing techniques. The results of the RCTs showed that the ultrasound-guided acupuncture and dry-needling treatment groups were relatively more effective than the control group in terms of cure rate, pain reduction, and functional improvement in the between-group comparison. This enhanced efficacy highlights the advantage of ultrasound-guided treatment, which allows for more precise targeting of invisible areas within the body and concentrated treatment directly at the lesion site, compared to traditional acupuncture techniques.

Case reports and case series have reported no adverse effects of ultrasound-guided acupuncture, thus confirming the safety and efficacy of ultrasound-guided acupuncture and dry-needling. Additionally, these techniques have been shown to be effective in improving symptoms and the function of the affected area.

In addition, clinical trials have compared the differences in patient perception of needle depth using ultrasound-guided acupuncture and dry-needling [86,87] and have analyzed the anatomical characteristics of the treatment area [88,89,90,91,92], the changes that occur inside the body after acupuncture treatment [93,94], needle displacement in typical acupuncture cases [95], the usefulness of ultrasound in acupuncture [96,97], the methodological aspects of the treatment technique [98], the efficacy of ultrasound-guided acupuncture and dry-needling [99,100,101,102], the safety and efficacy of conventional acupuncture procedures [103,104,105,106], disease mechanisms [107], and new treatment techniques [108]. This indicates that ultrasound-guided acupuncture and dry-needling are not only valuable as treatment modalities but also, in addition to enhancing conventional methods, serve as a foundation for developing new clinical procedures based on existing practices that can be applied to patients.

In these studies, ultrasound-guided acupuncture, dry-needling, acupotomy, pharmacopuncture, and embedding needling were performed alone and in combination with various modalities, such as high-energy shock wave therapy [20,32], physical therapy [45,46], joint mobilization therapy [25], and Chinese herbal prescriptions [51], with good therapeutic effects. These findings demonstrate that existing procedures in medical institutions can be expanded in multiple directions, highlighting the potential for these techniques to offer diverse treatment options for clinicians and be applied effectively to patients.

## 5. Limitations

This scoping study was limited by the fact that the types of studies included were mainly non-comparative, and there was a lack of systematic reviews and meta-analyses. By May 2024, one systematic review [15] and three meta-analyses [16,17,18] had been published, while three protocols for studies had been published, which is not a large number in absolute terms. Larger, more formal studies are needed, but few have been conducted in Korean medical centers, where acupuncture is commonly used. In addition, international studies may differ in terminology [8] or technique, and the interventions in these studies may differ from commonly used acupuncture and dry-needle treatments. Therefore, more high-quality multicenter clinical trials with larger sample sizes are needed to examine the efficacy and safety of ultrasound-guided acupuncture more closely. Second, this study did not include studies wherein ultrasound was only used to diagnose disease; therefore, the results may represent only a small portion of the quality of treatment with ultrasound devices used in real-world clinical practice, which may not represent the full range of applications of ultrasound devices in TCM clinics. Third, the researchers in the included studies did not have the same level of experience, and the ultrasound devices used in the studies were not uniform; therefore, the over or understatement of the results of the pre- and post-treatment processes cannot be excluded.

Nevertheless, this study is significant, since it is the first scoping review to extensively examine the entire spectrum of ultrasound-guided acupuncture treatments. It also provides a baseline for future researchers and clinical practitioners who wish to implement ultrasound-guided acupuncture in healthcare settings, by presenting the clinical conditions and symptoms for which ultrasound-guided acupuncture can be applied, specific methods of application, their effects, and various numerical outcome indicators that can be used to evaluate them. In particular, this study suggests practical applications of various techniques such as acupotomy, pharmacopuncture, and embedding needle.

## 6. Suggestions for Further Research

As such, this study provides two suggestions for future research on the clinical application of ultrasound-guided acupuncture. The first is to conduct research on various topics that can be applied to ultrasound in acupuncture and dry-needle therapy. This study was a scoping review only of articles that used ultrasound guidance for acupuncture and dry-needling, but a search of the database revealed various studies that used ultrasound guidance for acupuncture and dry-needling, including the diagnosis of diseases, evaluation, and comparison of treatment processes. Further research on this topic will expand the possibilities of combining ultrasound with acupuncture and dry-needling in healthcare organizations. Second, ultrasound-guided acupuncture treatments should be standardized. Currently, in the specific method of acupuncture treatment, the terminology is confusing, and the application of acupuncture varies depending on the discretion of the practitioner. Therefore, it would be beneficial for clinical researchers and practitioners to conduct standardized studies on the various aspects of ultrasound-guided acupuncture, such as the use of ultrasound devices.

## 7. Conclusions

Using a scoping review, this study analyzed 107 studies of ultrasound-guided acupuncture published through May 2024 and arrived at the following conclusions.

1. Research on ultrasound-guided acupuncture and dry-needling has been the subject of increasing interest. Various types of studies were conducted, and non-comparative studies were the most common with a total count of 47 (43.9%) comprised of 24 clinical trials, 14 case reports, and 9 case series; RCTs were the most common, with 41 (38.3%) as a single study design.
2. After categorizing diseases based on ICD-11, ultrasound-guided acupuncture and dry-needling is a reliable and effective treatment technique that can be applied to a variety of diseases. Among the total disease classifications of the papers included in the study, 48 (55.8%) were for diseases of the musculoskeletal system or connective tissue, and the most common sites were the shoulders and knees.
3. The application of ultrasound-guided acupuncture and dry-needling techniques varied depending on the research objectives and the conditions being treated, either as a standalone procedure or as a combined treatment.
4. Additional studies that confirm the effectiveness, statistical significance, and safety of ultrasound-guided acupuncture and dry-needling would provide a concrete evidence base for the use of ultrasound-guided acupuncture and dry-needling as stand-alone or combined treatment modalities in healthcare organizations.

## Figures and Tables

**Table 1:** Study design of selected studies.

Study Design	N (%)
Randomized controlled trial	41 (38.3%)
Non-randomized controlled trial	3 (2.8%)
Review	4 (3.7%)
Systematic review	1 (0.9%)
Meta-analysis	3 (2.8%)
Non-comparative study	47 (43.9%)
Cross-sectional study	4 (3.7%)
Prospective cohort study	2 (1.9%)
Pilot study	1 (0.9%)
Reliability study	1 (0.9%)
Total	107

**Table 2:** The Information of included RCT and nRCT.

Study ID	Study Type	Participant Count (Intervention/Control)	Intervention	Control	Target Disease	Outcome	Main Result
Ding Y (2013) [33]	RCT	n = 80 (20/20/20/20)	A: Interventional ultrasound combined with acupotomy	B: Conventional acupotomy	Shoulder joint disorder	VAS, CMC-Murley Shoulder Function Evaluation Scale	A: 51.85 ± 9.56→91.25 ± 5.75, (*p* < 0.01) B: 52.28 ± 7.96→75.72 ± 8.56, (*p* < 0.01)
					Knee osteoarthritis	VAS, HSS scale	A: 42.70 ± 9.19→90.40 ± 7.35, (*p* < 0.01) B: 43.23 ± 7.56→75.54 ± 9.21, (*p* < 0.01)
					Lumbar disc herniation	VAS, M-JOA scoring table	No statistically significant effect on the results
					Cervical disc herniation	VAS, Cervical Function Assessment Form	
Duan H (2016) [34]	RCT	n = 234, 117/117	A: Ultrasound-guided acupotomy group	B: Traditional knife group	Plantar fasciitis (16.98 ± 8.99 months)	VAS	B > A (*p* < 0.05).
						Tenderness score	B > A (*p* < 0.05).
						AOFAS-AH score	A > B (*p* < 0.05).
Chen T (2021) [49]	RCT	n = 70 (35/35)	A: Shallow-tissue thread embedding group	B: Deep-tissue thread embedding group	Obesity	Body mass, BMI, waist circumference, hip circumference	BMI and waist circumference: A > B (*p* < 0.05) Distention and fullness sensation and needling sensation and intensity: A > B (*p* < 0.05).
Zhang WB (2019) [43]	RCT	n = 74 (37/37)	A: Treated with ultrasound-guided intrathecal injection + releasing method of needle knife	B: Treated with ultrasound-guided intrathecal injection	Trigger finger	Self-made 9-score scale	Excellent/good rate: A > B (*p* < 0.05) Cure rate: A: 100.0%→97.3%, B: 13.5%→10.8%
Lan X (2023) [19]	RCT	n = 72 (42/30)	A: SNK group	B: OS group	Patients with grade 2 and above trigger digits	VAS, QG	VAS, QG of both groups decreased significantly.
Cao XY (2019) [44]	RCT	n = 36 (18/18)	A: ESWT + USGAP	B: ESWT Group	Frozen shoulder	NRS	A: 6.5→2.7, B: 6.7→4.3, A < B (*p* < 0.01).
						SPADI	A: 54.9→30.4, B: 56.1→43.2, A < B (*p* < 0.01).
Bubnov RV (2013) [35]	RCT	n = 133 (91/42)	A: US guidance group	B: Conventional dry-needling group (without US guidance)	Myofascial pain	VAS	A: 7.2→1.1 (pain reduced by 84%) (*p* < 0.001) B: 7.4→2.7 (pain reduced by 63.5%) (*p* < 0.001)
						Number of needles used in treatment	A: 2.6–0.54, B: 4.45–0.7
						Number of times a muscle twitch response was induced	A: 92.26–3.8%, B: 58.8–7.5%
						Number of TrP session	A: 2.3, B: 1.7–3.6
Bubnov R (2019) [20]	RCT	n = 40	A: Dry-needling under ultrasound guidance Group	B: High-energy extracorporeal shockwave therapy group	Chronic low back pain	VAS	A: 7.4→2.3, B: 7.2→5.2, but then recurred (*p* < 0.05).
Liu JY (2019) [36]	RCT	n = 52 (26/26)	A: Ultrasound-guided acupoint electrical stimulation group	B: Conventional acupoint electrical stimulation group	Ventilator-induced diaphragmatic dysfunction	Mechanical ventilation time	A < B (*p* < 0.05).
						Intensive care unit time	A ≓ B (*p* > 0.05).
						Total hospitalization time	A ≓ B (*p* > 0.05).
						Hospital mortality rate	A ≓ B (*p* > 0.05).
						Reintubation rate	A < B (*p* < 0.05).
Ding Y (2016) [21]	RCT	n = 60 (NA/NA)	A: Ultrasound-guided acupotomy group	B: Electro- acupuncture (EA) group	Knee osteoarthritis (KOA)	ADL	ADL: A > B (*p* < 0.01).
						HSS	A: 58 ± 5→86 ± 5, B: 60 ± 4→77 ± 6 A > B (*p* < 0.01).
						VAS	A: 5.1 ± 1.1→2.1 ± 1.7, B: 5.1 ± 1.5→3.1 ± 1.2 A < B (*p* < 0.01)
						Degree of infrared thermal images	A: 0.81 ± 0.21→0.32 ± 0.12, B: 0.78 ± 0.25→0.33 ± 0.14 A ≓ B (*p* < 0.01).
Sun W (2015) [50]	RCT	n = 90 (30/30/30)	A: Deep-layer embedding group (multifidus muscle layer)	B: Middle-layer embedding group (semispinalis capitis muscle layer) C: Shallow-layer embedding group (subcutaneous layer),	Cervical spondylosis	Symptoms and functional score	Significantly increased in A (compared to B, C. Both *p* < 0.05).
						PRI, VAS, PPI	Significantly decreased after treatment in A and B compared to C (all *p* < 0.05).
						NDI	A and B decreased after treatment (*p* < 0.05), A decreased significantly compared to B,C (all *p* < 0.05).
Sharif F (2023) [45]	RCT	n = 96 (48/48)	A: UG-DN + CPT (conventional physical therapy) Group	B: CPT group	Jumper’s knee	VAS	A: 8 ± 0.00→3 ± 1, B: 8 ± 2→1.5 ± 1, A > B (*p* = 0.000).
						VISA-p questionnaire	A: 52 ± 8→83.5 ± 7, B: 51 ± 12→92 ± 2, A < B (*p* = 0.000).
						Lysholm scale	A: 67 ± 3→84 ± 5, B: 65 ± 8→92 ± 4, A < B (*p* = 0.000).
						KOOS	A: 52.5 ± 8→83.5 ± 8, B: 52 ± 12→92 ± 3, A < B (*p* = 0.000).
Benito-de-Pedro AI (2023) [22]	RCT	n = 52 (26/26)	A: Deep dry-needling (DDN) group	B: Percutaneous electrolysis (PE) group	Active myofascial trigger points of the levator scapulae	Pain intensity	A: 6.80 ± 1.13→3.00 ± 2.04, B: 6.77 ± 1.03→2.77 ± 2.29 A ≓ B (*p* > 0.05)
						PPT (at central MTrP)	A: 2.52 ± 0.70→2.74 ± 0.81, B: 2.56 ± 0.75→2.72 ± 0.77 A ≓ B (*p* > 0.05).
						Cervical ROM	A: 68.79 ± 8.50→74.13 ± 4.97, B: 72.06 ± 4.04→75.13 ± 3.84 A < B (*p* < 0.05).
						Neck disability	A < B (*p* = 0.047).
						Post-needling soreness	A ≓ B (*p* > 0.05).
Sharif F (2022) [46]	RCT	n = 94 (47/47)	A: Ultrasound-guided dry-needling + CPT	B: CPT group	Jumper’s knee	VAS	A: 8.20 ± 0.75→3.00 ± 1.43, B: 8.00 ± 0.64→6.40 ± 0.49 A < B ((*p* = 0.000).
						VISA-p	A: 43.10 ± 7.08→88.70 ± 8.59, B: 40.70 ± 6.61→78.20 ± 8.34 A > B (*p* = 0.000).
						Lysholm scale	A: 56.00 ± 4.05→83.40 ± 5.78, B: 55.20 ± 2.16→80.20 ± 4.54 A > B (*p* = 0.000).
						KOOS	A: 56.56 ± 7.12→89.29 ± 5.19, B: 51.29 ± 7.6→79.40 ± 6.19 A > B (*p* = 0.000).
Qin X (2022) [23]	RCT	n = 68 (34/34)	A: Ultrasound-guided 18G-PTC puncture needle group	B: Small needle knife therapy group	Primary frozen shoulder	Overall efficacy	A: 88.23%, B: 67.64%, A > B (*p* < 0.05)
						UCLA scores of the shoulder joint	A: 13.61 ± 3.77→31.22 ± 3.34, B: 14.34 ± 3.89→25.43 ± 3.83 A > B (*p* < 0.05)
						shoulder mobility	A > B (*p* < 0.05)
						muscle elasticity and thickness	A > B (*p* < 0.05)
						VAS	A: 7.12 ± 1.44→3.41 ± 1.39, B: 7.21 ± 1.45→5.02 ± 1.76 (*p* > 0.05)
Shen Y (2022) [37]	RCT	n = 59 (NA/NA)	A: Operated with US guidance	B: Operated without US guidance	De Quervain’s disease	Regarding the amount of release	A: 20 cases (87%) vs. B: 27 cases (75%)
Wang YH (2023) [51]	RCT	n = 60 (NA/NA)	A: Received juanbi decoction 3 times daily for 2 weeks along with an acupotomy assisted by ultrasound	B: Same protocol was used with the group A, but the juanbi decoction was replaced with normal saline	Lumbar disc herniation	VAS	A: 4.87 ± 0.57→3.67 ± 0.48, B: 4.8 ± 0.61→3.43 ± 0.73 A ≓ B (*p* < 0.05)
						ODI	A: 66.20 ± 2.91→50.93 ± 5.79, B: 66.8 ± 4.80→47.4 ± 6.22 A > B (*p* < 0.05)
						LBOS	A: 24.57 ± 2.67→29.4 ± 3.94, B: 24.06 ± 2.24→30.67 ± 4.96 A ≓ B (*p* < 0.05)
						JOA	A: 11.23 ± 1.61→16.83 ± 2.60, B: 11.43 ± 1.65→18.67 ± 1.79 A < B (*p* < 0.05)
Zhang S (2019) [47]	RCT	n = 51 (NA/NA)	A: Steroid injection combined with ultrasound-guided MSN release group	B: Steroid injection group	Carpal tunnel syndrome (CTS)	BCTQ-SSS	A: 3.10 ± 0.32→1.84 ± 0.21 (*p* = 0.096) B: 3.00 ± 0.25→2.06 ± 0.23 (*p* < 0.001)
						BCTQ-FSS	A: 3.10 ± 0.25→1.80 ± 0.35 (*p* = 0.112) B: 3.00 ± 0.25→2.08 ± 0.27 (*p* < 0.001)
						CMAP	A: 9.4 ± 1.2→12.2 ± 1.3 (*p* = 0.613) B: 9.5 ± 1.1→11.3 ± 1.1 (*p* < 0.001)
						DML	A: 5.2 ± 0.3→4.5 ± 0.4 (*p* = 0.002) B: 5.4 ± 0.3→4.7 ± 0.4 (*p* < 0.001)
						SNAP	A: 12.1 ± 1.8→16.3 ± 3.5 (*p* = 0.368) B: 12.0 ± 1.6→15.4 ± 2.7 (*p* < 0.001)
						SNCV	A: 38.6 ± 3.8→46.5 ± 2.5 (*p* < 0.597) B: 39.5 ± 3.2→44.7 ± 3.2 (*p* < 0.001)
						CSA	A: 13.3 ± 1.4→10.8 ± 1.1 (*p* = 0.493) B: 13.1 ± 1.5→11.6 ± 1.2 (*p* < 0.001)
Bureau NJ (2022) [24]	RCT	n = 62 (NA/NA)	A: Dry-needling (US guieded)	B: Surgery	Chronic lateral epicondylosis	PRTEE score	B 33.4 (CI 25.2–41.5) > A 26.9 (CI 19.4–34.4) (*p* = 0.25).
						Proportion of successful treatment	B 83% (CI 63–95%) > A 81% (CI 63–93%) (*p* = 1.00).
Bubnov R (2011) [38]	RCT	N = 133 (91/42)	A: Dry-needling (US guieded)	B: Dry-needling	MPS (myofascial pain syndrome)	Pain relief effect and level of inducing local twitch response (LTR)	Increased in A
						Average number of needling trigger points, average number of treatment sessions	Decreased in A
Bubnov RV (2015) [52]	RCT	n = 32 (NA/NA)	A: Received dry-needling (DN) of paravertebral (“central”) MTrP under ultrasound guidance	B: Received DN under ultrasound guidance of “peripheral” MTrP in muscles	LBP (lower back pain)	VAS	A: 7.2→1.2, B: 7.3→3.5, A < B (*p* < 0.05)
						PainDetects (1–38) scores	A: 98% (18.3→9.2), B: 25% (18.5→11.5), A > B (*p* < 0.01)
						MTrP recurrence	A: 25%, B: 58% (*p* < 0.01) at 24 h after manipulation; outcome at 7th day was A: 7%, B: 35% (*p* < 0.05).
Samiei SM (2021) [25]	RCT	n = 34 (NA/NA)	A: Ultrasound-guided dry-needling with Mulligan mobilization technique (DN with MM)	B: Only dry-needling (DN) C: Received no intervention	Lateral epicondylitis	Pain intensity, Function level	A, B had a significant improvement compared to C. Function and VAS scores: A > B
						Tendon Thickness of extensor muscles	A ≓ B
De Boer FA (2017) [26]	RCT	n = 25 (NA/NA)	A: Dry-needling (US guieded)	B: Radial Shockwave (RSWT)	Shoulder calcific tendinitis	NRS	A: 7.5→1.9, B: 7.9→2.1
						Oxford	A: 38.5→53.2, B: 38.5→49.1
Zhu Ting (2018) [61]	RCT	n = 52 (26/26)	A: Drug injection and acupotomy (US guieded)	B: Drug injection and cupotomy (under the guidance of palpation)	De Quervain’s disease	VAS, Quinnell scoring	A < B (all *p* < 0.05)
Tabatabaiee A (2019) [62]	RCT	n = 32 (16/16)	A: Dry-needling (US guieded) + Advice	B: Waitlist control group (Only advice)	PMS	ODI, PPT, transverse-plane hip ROM	A < B (*p* = 0.007).
Xie N (2019) [27]	RCT	n = 48 (24/24)	A: Ultrasound-guided dry-needling for myofascial trigger points + with stretching training	B: Sole non-weight-bearing plantar fascia stretching	Plantar fasciitis	NPRS, AOFAS, PCS, MCS, SF-36	The overall differences of NPRS, AOFAS, PCS and MCS were significant before and after treatment in both two groups (all *p* = 0.05).
Huang Y (2022) [28]	RCT	n = 54 (28/26)	A: Dry-needling (ultrasound-guided)	B: Pharmacotherapeutic group	Post-therpetic neuralgia mixed with myofascial pain syndrome	VAS, MPQ	Effective rate: 92.9% vs. 38.5% (A vs. B) (*p* < 0.01) Recurrent rate: 7.1% vs. 34.6% (A vs. B) (*p* = 0.02) Satisfactory rate: A > B
Pang JCY (2022) [29]	RCT	n = 84 (28/28/28)	A: Dry-needling (ultrasound-guided) + exercise	B: Placebo ultrasound-guided DN with exercise C: exercise therapy solely	Knee osteoarthritis	VAS,	A is better compared to B and C A vs. B: MD = −15.61, 95% CI [−25.49, −5.51], (*p* = 0.001) A vs. C: MD = −19.90, 95% CI [−29.71, −10.08], (*p* < 0.001).
						KOOS-pain,	A is better compared to B and C
						KOOS-symptoms, KOOS-quality-of-life	not statistically significant between groups.
Jin HP (2022) [30]	RCT	n = 120 (40/40/40)	A: Ultrasound-guided EA (electro-acupuncture) at suprhyoid muscle	B: EA at CV23, GB12, GB20, etc. C: suprahyoid muscle according to anatomical location	Pharyngeal dysphagia after stroke	PAS score	PAS score: A < B, C (*p* < 0.05)
						IF scores	A > B, C (*p* < 0.05).
						Forward and upward movement distance of hyoid bone and thyroid carthilage	A > B, C (*p* < 0.05).
						Incidence of subcutaneous hematoma	A 0% (0/40) < B 20.0% (8/40) < C 47.5% (19/40) (*p* < 0.05).
Xu H (2022) [48]	RCT	n = 63 (33/30)	A: Ultrasound-guided hydrodilatation of glenohumeral joint combined with acupotomy	B: Only treated with ultrasound-guided hydrodilatation of glenohumeral joint)	Frozen shoulder	Active ROM	A > B (all *p*< 0.05)
						CMS score	
						CHL thickness	A < B (all *p*< 0.05)
						Rate of hypoecoic thickening in rotator cuff space	
Zheng Y (2014) [53]	RCT	n = 169 (NA/NA)	A: UG-MSN	B: UG-DN	Chronic neck pain	VAS, PCS, MCS	VAS: A < B (both *p* < 0.0001). A also showed significantly lower scores on the adjusted neck disability index and PCS
Krasny C (2005) [32]	RCT	n = 80 (40/40)	A: Needling (ultrasound-guided) + high-energy shockwave therapy	B: High-energy shockwave therapy	Calcific tendonitnis	Pain	A: 6.7 ± 2.6→13.3 ± 3.7, B: 5.6 ± 2.3→10.6 ± 4.1 (*p* < 0.001)
						Daily activity	A: 11.0 ± 3.3→18.1 ± 4.2, B: 10.7 ± 3.0→16.0 ± 3.9 (*p* < 0.001)
						Movement	A: 19.1 ± 6.4→32.7 ± 9.8, B: 19.2 ± 6.4→29.7 ± 10.1 (*p* < 0.001)
						Power	A: 9.5 ± 4.4→12.7 ± 4.2, B: 8.7 ± 3.5→11.0 ± 5.2 (*p* < 0.001)
Pan M (2019) [39]	RCT	n = 41 (20/21)	A: Needle knife (US guieded)	B: Needle knife (blind release)	Trigger finger	Clinical grade	A: Grade 0: 0→20/Grade 1: 0→0/Grade 2: 2→0/Grade 3: 10→0/Grade 4: 8→0 B: Grade 0: 0→4/Grade 1: 0→15/Grade 2: 2→0/Grade 3: 8→1/Grade 4: 11→1
						Complications	No any complications had been happened in the A group.
						Operation time	A: 15.21 ± 0.87 min, B: 5.23 ± 0.55 min. A > B (*p* < 0.05)
Zhou Q (2023) [40]	RCT	n = 100 (NA/NA)	A: Acupotomy (ultrasound-guided)	B: Acupotomy (non ultrasound-guided)	Anatomical study	Injury rate	A 0% vs. B 6%, 12%, 20% (the rate of nerve, blood vessel and tendon damage) (*p* < 0.05)
						Width of the transverse carpal ligament	A 86% vs. B 36% (PL < 0.05)
Qiu Z (2022) [41]	RCT	n = 84 (28/28/28)	A: Ultrasound-guided needle knife pushing group	B: Non-ultrasound-guided needle knife pushing group C: classical needle knife operation puncture group	A1 pulley release	Relevant anatomical data	Injured cases: A 29 (20.7%)/B 36 (25.7%)/C 28 (20.0%)
							Missed release cases: A 8 (5.7%)/B 4 (2.9%)/T 13 (9.3%)
							Percentage of released A1 pulley: A 71.4% ± 30.7%/B 66.0% ± 20.3%/C 61.0% ± 30.4%
							Full release rates of the groups: A (31.4%) > B (15.7%) > C (13.6%)
Lin S (2024) [54]	RCT	n = 100 (50/50)	A: Regular acupuncture (ultrasound-guided)	B: Shallow acupuncture (ultrasound-guided)	C hronic subjective dizziness (CSD)	Clinical effectiveness	A (94%) > B (80%) (*p* = 0.037)
						PSQI	A: 15.37 ± 7.82→6.83± 3.65, B: 15.98 ± 10.83→8.18 ± 4.05 A < B (*p* < 0.05)
						DHI	A: 51.37 ± 16.89→32.73 ± 5.41, B: 50.65 ± 15.81→37.81 ± 7.52 A < B (*p* < 0.05)
						HAMD	A: 15.36 ± 7.18→5.87 ± 3.26, B: 15.51 ± 7.82→7.84 ± 3.98 A < B (*p* < 0.05)
						F atigue Severity Scale (FSS)	A: 33.48 ± 13.78→14.96 ± 6.98, B: 33.92 ± 14.05→18.23 ± 8.62 A < B (*p* < 0.05)
						HAMA	A: 21.65 ± 11.72→7.96 ± 4.81, B: 21.23 ± 11.54→11.78 ± 5.98 A < B (*p* <0.05)
Wang (2023) [55]	RCT	n = 106 (53/53)	A: Musculoskeletal ultrasound-guided acupuncture	B: Conventional ultrasound-guided acupuncture	Osteoarthritis	VAS	A (4.3 ± 0.7) < B (*p* < 0.05)
						Lysholm scale	A < B (*p* < 0.05) support scores: not different between two groups (*p* > 0.05)
Dai J (2023) [56]	RCT	n = 74 (37/37)	A: Deep acupuncture group (ultrasound-guided)	B: Shallow acupuncture group (ultrasound-guided)	N/A (bladder in controlling urine)	PSV	A: 39.96→52.55, B: 41.50→47.55 A >B (*p* < 0.05)
						TAMX	A: 8.63→12.54, B: 10.13→10.95 A > B (*p* < 0.05)
						EDV	A: 2.26→2.34, B: 1.56→1.63 A > B (*p* < 0.05)
						PI	A: 5.62→4.99, B: 4.59→4.57 A > B (*p* < 0.05)
						RI	A: 0.95→0.95, B: 0.97→0.95 A ≓ B (*p* < 0.05)
						Bladder volume	A: 25.27→50.70, B: 30.56→40.48 A > B (*p* < 0.05)
						C-MASS	A: 42.30, B: 9.03, A > B (*p* < 0.01)
Guner D (2023) [31]	RCT	n = 44 (22/22)	A: Ultrasound-guided dry-needling group	B: Physical exercise treatment group	PMS	VAS	A: 7.6 ± 1.6→2.5 ± 2.1, B: 7.8 ± 0.7→2.6 ± 1.1 A ≓ B (*p* > 0.05)
						ODI	A: 20.9 ± 8.5→7.4 ± 5.7, B: 32.6 ± 6.7→9.9 ± 7.7 A ≓ B (*p* > 0.05)
						LEFS	A: 41.7 ± 15.9→69.4 ± 12.9, B: 42.4 ± 15.8→71.3 ± 8.2 A ≓ B (*p* > 0.05)
						DN4	A: 3.5 ± 2.1→0.9 ± 1.2, B: 4.1 ± 2.6→1.3 ± 1.4 A ≓ B (*p* > 0.05)
Zhu (2024) [42]	RCT	n = 70 (35/35)	A: Ultrasound-guided group	B: Without ultrasound guiding group	Lumbar disc herniation	VAS	A: 5.49 ± 1.01→0.57 ± 0.61, B: 5.23 ± 1.03→1.86 ±1.03 A < B (*p* < 0.01)
						ODI)	A: 43.91 ± 10.02→5.71 ± 8.40, B: 41.17 ± 13.00→24.86 ± 14.35 A < B (*p* < 0.01)
						JOA	A: 15.43 ± 2.21→25.37 ± 2.95, B: 16.74 ± 2.65→22.86 ± 2.52 A > B (*p* < 0.01)
						MOS SF-36	A: 75.54 ± 8.22→83.97 ± 11.79, B: 71.83 ± 8.07→79.31 ± 12.12 A > B (*p* > 0.05)
Pu J (2023) [57]	RCT	n = 160 (80/80)	A: Ultrasound-guided injection acupotomy	B: Ultrasound-guided SNRB	Cervical spondylotic radiculopathy (CSR)	Odom’s criteria clinical curative effect	A: 93.6% vs. B: 81.0%, A > B (*p* = 0.018)
						VAS	A: 6.1→1.0, B: 6.3→1.8 A < B (*p* = 0.03)
						NDI	A: 51.4 ± 13.3→15.4 ± 12.8, B: 51.8 ± 13.0→21.9 ± 16.2 A > B (*p* = 0.006)
						SF-36	A: 43.3 ± 17.5→80.1 ± 12.6, B: 44.0 ± 16.5→72.6 ± 19.1 A > B (*p* = 0.004)
Lin Q (2005) [58]	nRCT	NA	A: Treatment group (electro-acupuncture with strong stimulation)	B: Medication C: Conventional acupuncture	Upper segment ureterolithiasis	Cure rate	A > B, C (*p* < 0.05)
						Total effective rate	A > B, C (*p* < 0.01)
Arias-Buría JL (2023) [59]	nRCT	n = 100 (50/50)	A: Ultrasound-guided group	B: Palpation-guided group	Anatomical study	Distance to the targeted	A: 0.25 ± 0.65 mm, B: 2.5 ± 1.9 mm A < B (*p* < 0.001)
						Time of the procedure	A: 54.8 ± 26.8 s, B: 23.75 ± 15.4 s A > B (*p* < 0.001)
						Accuraterate of insertions	A: 100%, B: 80% A > B
						Tissue number of passes	A: 2.55 ± 1.9, B: 1.5 ± 0.95, A > B (*p* = 0.001)
						Unintentional puncture structures	A: 16%, B: 52% A < B (*p* < 0.001)
Malo-Urriés M (2024) [60]	nRCT	n = 100 (50/50)	A: Ultrasound-guided group	B: Palpation-guided group	Anatomical study	Distance to interface	A: 0.2 ± 0.7, B: 3.5 ± 2.2 A < B (*p* < 0.001)
						Longitudinal contact of the needle	A: 5.3 ± 2.2, B: 0.6 ± 1.8 A > B (*p* < 0.001)
						Time required	A: 53.8 ± 18.9, B: 19.1 ± 6.5 A > B (*p* < 0.001)
						Tissue number of passes	A: 2.8 ± 1.5, B: 1.7± 0.9 A > B (*p* < 0.001)
						Unintentional puncture structures	A: 5 (10%)mB: 9 (18%) A < B (*p* = 0.249)

Notes. NA: not available; VAS: Visual Analog Scale; HSS: Hospital Special Surgery Index; M-JOA: Modified Lumbago Assessment by Japanese Orthopedic Association; AOFAS-AH: American Orthopedic Foot And Ankle Society Ankle–Hindfoot Scoring System; BMI: body mass index; SNK: small-needle knife; OS: open surgery; QG: Quinnell grading; ESWT: extracorporeal shock wave; USGAP: ultrasound-guided acupotomy; NRS: numeral rating scale; SPADI: Shoulder Pain and Disability Index; US: ultrasound; DN: dry-needling; TrP: trigger point; MTrP: myofascial trigger point; ADL: activities of daily living; PRI: Pain Rating Index; PPI: Present Pain Index; NDI: Neck Disability Index; CPT: conventional physical therapy; VISA-P: Victoria Institute of Sports Assessment—Patellar Tendinopathy; KOOS: Knee Injury and Osteoarthritis Outcome Score; PPT: pressure pain threshold; ROM: range of motion; JOA: Japanese Orthopedic Association; ODI: Oswestry Disability Index; LBOS: Lower Back Pain Outcome Scale; MSN: miniscalpel needle; BCTQ: Boston Carpal Tunnel Questionnaire; SSS: Symptom Severity Scale; FSS: Functional Status Scale; CSA: cross-sectional area; DML: distal motor latency; CMAP: compound muscle action potential; SNAP: sensory nerve action potential; SNCV: sensory nerve conduction velocity; PRTEE: patient rated tennis elbow evaluation; NPRS: numeric pain rating scale; PMS: priformis muscle syndrome; MCS: Mental Composite Score; PCS: Physical Composite Score; SF-36: Short-Form 36 Health Survey; MPQ: McGill Pain Questionnaire; PAS: Penetration Aspiration Scale; IF: Ichiro Fujima Ingestion Swallowing Functions Score; CMS: Constant–Murley scale; CHL: coracohumeral ligament; UG-MSN: ultrasound-guided Miniscalpel needle release; UG-DN: ultrasound-guided dry-needling; PSQI: Pittsburgh Sleep Volume; DHI: Dizziness Handicap Inventory; HAMD: Hamilton Depression Scale; HAMA: Hamilton Anxiety Scale; PSV: peak systolic velocity; TAMX: time average maximum velocity; EDV: end diastolic velocity; PI: Pulsatility Index; RI: Resistance Index; LEFS: Lower Extremity Functional Scale; DN4: Douleur Neuropathique 4 Questionnare score; SNRB: selective nerve root block.

**Table 3:** Information regarding included case reports and case series.

Study ID	Intervention Method		Number of Case (Male/Female)			Target Disease	Treatment Point	Outcome	Main Result	
Bağcıer F (2020) [63]	Dry-needling (ultrasound-guided, 0.60 × 60 mm)		1/0			PMS	Piriformis MTrP	VAS	7→2	
								ODI	78→35	
Zenita Y (2018) [64]	Dry-needling (ultrasound-guided fascia release DN)		1/0			Mandibular numbness	1 cm bone margin anterior to mandibular angle	VAS	80/100→0/100	
								US image’s intensity	Decreased	
								Mouth opening (dental arch width)	3 cm→5 cm	
Fusco P (2021) [65]	Dry-needling (ultrasound-guided, size n° 8,)		1/NA			Adductor injury (mofascial pain)	Left thigh adductor	VAS	VAS 6→2→0	
								Elastosonography sign	restored to blue→red	
Song XZ (2022) [80]	Electroacupuncture (ultrasound-guided, 30 × 40 mm) + training (spinal joint loosening training, respiratory training, lumbar comprehensive sports training, paraplegic limbs comprehensive training) + manipulative treatment		0/1			SPI	Sacral nerve: Both S3, S4 foramen (30 × 75 mm, 20 Hz, 220 ms wave width, 100–120 mV. 1 h)	ASIA scale (motor, sensory score)	Before sacral stimulation: 58→after sacral stimulation: 64→after addition of spinal and cranial stimulation: 73 sensory score increased to 150→160→186	
							Spinal cord: 2 between T12 and L1, 2 on the left and right nerve roots of L1, 2 between L1 and 2 (30 × 40 mm, 50 Hz, 220 ms wave width, 1 h)	FIM	67→81 (After sacral stimulation)→114 (After spinal cord and skull stimulation)	
							Head: in front of the central sulcus and extended along with the skull to the front of the forehead (1.5 cm)	All symptoms	Symptoms of functional remission (decreased urinary retention) were observed.	
Rao Y (2022) [84]	Perineural injection (ultrasound-guided, 0.4% lidocaine) + Acupotomy (0.4 × 40 mm)		1/0			IBSN as a complication of ACLR	NA	VAS	10 to 1	
								diameter of the IBSN	Smaller	
								peripheral soft tissue signal	Hypoechoic on ultrasound	
Settergren R (2013) [66]	Dry-needling (ultrasound-guided, 0.50 × 75 mm) + Therapeutic exercise		0/1			Supraspinatus tendinopathy	Pathologic tissue of supraspinatus tendon	Symptom	Full resolution of symptoms.	
								ROM	Eliminate ROM limitations	
Jia Y (2020) [67]	Dry-needling (ultrasound-guided, 0.35 × 60 mm)		1/0			Vasomotor rhinitis (VMR)	Sphenopalatine ganglion	Symptom	Frequency of sneezing episodes was reduced in the morning, the nasal congestion and runny nose were relieved	
Kurosawa A (2019) [68]	Dry-needling (ultrasound-guided)		0/1			Right shoulder pain	Fascia between the deltoid muscle and the supraspinatus tendon	NRS	8→2	
								active-ROM	Abduction: 70→120 degrees Extension: 30→45 degrees	
Afonso J (2023) [81]	Electroacupuncture (ultrasound-guided, 4 Hz, 100 ms, 20 min)		1/0			Thoracic myofascial pain syndrome	Palpable painful and taut band (muscle layers along the medial border of the scapula, specifically the trapezius, rhomboid, and erector spinae muscles)	NRS	7→3	
								ROM, Sleep	Improved ROM and sleep	
								Mood	Mood does not improved	
Pai RS (2018) [69]	Dry-needling (ultrasound-guided, 32 gauge needle)		0/1			CRPS-1(right upper limb)—pronation deformity and myofascial issues around the shoulder	Muscles of the neck, shoulders, arms, forearms, and hands	DASH score	88.8→33.5 (3 months)→10.3 (1 year)	
								Pain Detect score	10→2 (3 months)→1 (1 year)	
								PHQ-9	15→100% recovery (3 months)→Maintenance (1 year)	
								ROM	Shoulders, hands: Limited→Almost complete (3 months)→Complete (1 year)	
								ROM	No improvement (radio-ulnar and humero-radial joint)	
Ou YY (2024) [85]	Ultrasound-guided acupotomy		1/0			CPNES	Peroneus longus muscle	Heaviness and numbness	greatly relieved	
								Two-point discrimination	0→25 mm	
Stewman CG (2023) [82]	Ultrasound-guided electroacupuncture		1/0			Rotator cuff (RTC) tendinopathy	Areas of RTC tendon injury	Patient states	85% improvement in symptoms, “minimal” residual discomfort with one stretching movement	
Temel MH (2024) [70]	Ultrasound-guided dry-needling		1/0			Chronic lower back pain (CLBP)	Quadratus lumborum (QL) muscle	VAS	7→3	
Hemani (2022) [71]	Ultrasound-guided dry-needling		0/1			Post laminectomy infective spondylodiscitis	Muscles of the back, thigh, calf, foot, and sole	VAS	8→2	
								Limit of standing	5~10 min→150 min	
								ESR	95→25	
								CRP	40→22.5	
								ODI	42→11	
								PFWD	0→70	
Malik D (2016) [72]	Dry-needling (ultrasound-guided)		120 (NA)			Achilles tendonitis, plantar fasciitis, and lateral epicondylitis	Affected site	Symptom (pain)	Showed symptom improvement in 80% of cases.	
Vas L (2023) [73]	Dry-needling (ultrasound-guided)		35 (13/22)	23		Trigeminal neuralgia	Masticatory muscles, facial muscles, neck muscles	NRS, medication discontinuition and dose	NRS 8.9→0.6 (after DN)	18 people discontinued medication, carbamazepine dose reducing from 716.7 mg/day to 113.0 mg/day.
	Dry-needling (ultrasound-guided) + PRF (pulsed radiofrequency)	Trigemnal gangalion (TG)		12	6				NRS 8.8→5.7 (after PRF)→1.0 (after DN)	
		mandibular nerve			6					
Fusco P (2018) [74]	Dry-needling (ultrasound-guided, 0.30 × 60 mm)		3 (1/2)			PMS	Piriformis muscle; gluteus minimus, medius, maximus	Symptom	Resolved.	
Parthasarathy S (2022) [83]	Electroacupuncture (ultrasound-guided)		2	1/0		Wrist drop	DU20/LI6,7,8/TW 6,7,8	Motor power of the extensors of the wrist	Improved from 1/5→3/5 to 4/5.	
				0/1		Tennis elbow	DU20/LI 4,10,11,12/Ashi points	VAS	7–8/10→2–3/10	
Vas L (2019) [75]	Dry-needling (ultrasound-guided)		4			PMPS	Muscles contributing to myofascial pain (neck, shoulder, chest wall and limb girdle, upper arm, forearm)	NRS	7.8→1.3	
								PD	20.0→6.6	
								DASH	61.0→22.5	
								PHQ-9	no improvement	
								opioid use		
	Dry-needling (ultrasound-guided) after neural interventions (NIs)		16					NRS	9.6→5.2 (after Nis)→2.3 (after DNs)	
								PD	28.3→16.1 (after Nis)→6.6 (after DN)	
								DASH	80.9→71.1 (after Nis)→34.6 (after DN)	
								PHQ-9	12 mild, 5 moderate, 3 severe→12 mild, 6 moderate after NIs→6 mild, 4 moderate, 10 No depression after DN	
								Medication	Morphine: 12→8 people discontinued, 2 people took only half dose Fentanyl patch: 9→2 people discontinued, 3 people took only half dose	
Bubnov RV (2010) [76]	Dry-needling (ultrasound-guided)		91			MPS	NA	NA	The use of US examination significantly improve the effectiveness and safety of DDN as an optimal method of inactivation of the trigger points.	
Kamble (2024) [77]	Ultrasound-guided dry-needling		30 (NA)			Soleus muscle spasticity in stroke survivors	Spastic soleus muscle	Thickness of the soleus muscle	8.88 ± 2.38→11.55 ± 2.60 (mm) (*p* < 0.001)	
								MMAS	3.0→1.53 (*p* < 0.001)	
								MTS	15.76 ± 2.22→18.46 ± 2.31 (*p* < 0.001)	
								H-reflex	2.74 ± 3.20→1.34 ±1.92 (*p* < 0.001)	
R. Bubnov (2023) [78]	Ultrasound-guided dry-needling		20 (NA)			Myofascial trigger points (MTrP)	Multifidus muscles	Muscle thickness	23.5–33.3% decreased	
								Muscle width	Not statistically significant	
								CSA	1.8 mm→0.9 mm	
								PA	17.2 degrees→8.7 degrees	
R. Bubnov (2023) [79]	Ultrasound-guided dry-needling		20 (20/0)			Combat injuries (myofascial and neuropathic pain)	Myofascial trigger points	VAS	Decreased	
								Fascicle diameter	2→0.9	

Notes. SPI: spinal cord injury; ASIA: American Spinal Injury Association; FIM: functional independence measure; IBSN: injuries to the infrapatellar branch of the saphenous nerve; ACLR: anterior cruciate ligament reconstruction; CRPS: complex regional pain syndrome; CPNES: common peroneal nerve entrapment syndrome; ESR: Erythrocyte sedimentation rate; CRP: C-reactive protein; PFWD: pain-free walk distance; DASH: disabilities of arm, shoulder, and hand; PHQ-9: patient health questionnaire-9; PMPS: postmastectomy pain syndrome; PD: pain detect; MPS: myofascial pain syndrome; MMAS: Modified Ashworth Scale; MTS: Modified Tardeau Scale; PA: pennation angle.

## Supplementary Materials

The following supporting information can be downloaded at: https://www.mdpi.com/article/10.3390/jcm13164962/s1, Appendix S1: Search Expressions (DOCX); Appendix S2: PRISMA ScR Checklist (DOCX); Appendix S3: Disease Categories Based on ICD-11 Classifications; Appendix S4: The information of included Systematic Review and Meta-analysis; Appendix S5: The information of included Clinical Trial; Appendix S6: The Information of included cohort study; Appendix S7: The Information of included RCT and nRCT.
