# Supplementary material for: Analysis of Research Trends in Ultrasound-Guided Acupuncture and Dry-Needling: A Scoping Review
Source: J Clin Med. 2024 Aug 22;13(16):4962. doi: 10.3390/jcm13164962 (PMC11355774; doi:10.3390/jcm13164962)
Supplement: Supplementary file 1 [file jcm-13-04962-s001.zip › Appendix S1. Search Expressions.pdf]

S1 Appendix. Search Expressions

1.1 Pubmed

#1 : "Ultrasonography" [Mesh] OR "echography" [TIAB] OR "diagnostic ultrasonic imaging" [TIAB] OR "diagnostic ultrasound" [TIAB] OR "doptone" [TIAB] OR "duplex echography" [TIAB] OR "echogram" [TIAB] OR "echographic evaluation" [TIAB] OR "echography" [TIAB] OR "echoscopy" [TIAB] OR "echosound" [TIAB] OR "high resolution echography" [TIAB] OR "sonogram" [TIAB] OR "sonographic examination" [TIAB] OR "sonographic screening" [TIAB] OR "sonography" [TIAB] OR "ultrasonic detection" [TIAB] OR "ultrasonic diagnosis" [TIAB] OR "ultrasonic echo" [TIAB] OR "ultrasonic examination" [TIAB] OR "ultrasonic scanning" [TIAB] OR "ultrasonogram" [TIAB] OR "ultrasonographic examination" [TIAB] OR "ultrasonographic screening" [TIAB] OR "ultrasonography" [TIAB] OR "ultrasound diagnosis" [TIAB] OR "ultrasound scanning" [TIAB] OR "ultrasound" [TIAB] OR "phonophoresis" [TIAB] OR "radiation" [TIAB] OR "ultrasonic" [TIAB] OR "sonication" [TIAB] OR "sonification" [TIAB] OR "ultra sound" [TIAB] OR "ultrasonic" [TIAB] OR "ultrasonic energy" [TIAB] OR "ultrasonic irradiation" [TIAB] OR "ultrasonic measurement" [TIAB] OR "ultrasonic sound" [TIAB] OR "ultrasonic wave" [TIAB] OR "ultrasonic waves" [TIAB] OR "ultrasonics" [TIAB] OR "ultrasound" [TIAB] OR "ultrasound radiation" [TIAB] OR "ultrasound-guided" [TIAB]
#2 : “acupuncture” [Mesh] OR “acupuncture” [TIAB] OR “acupuncture therapy” [TIAB] OR “shonishin” [TIAB] OR “dry needling” [TIAB] OR “dry needle technique” [TIAB] OR “dry needle therapy” [TIAB] OR “intramuscular stimulation” [TIAB] OR “muscle dry needling” [TIAB] OR “myofascial trigger point dry needling” [TIAB] OR “trigger point dry needling” [TIAB] OR “needling” [TIAB] OR “electroacupuncture” [TIAB] OR “electric acupuncture” [TIAB] OR “electrical acupoint stimulation” [TIAB] OR “electrical acupuncture” [TIAB] OR “electro-acupuncture” [TIAB] OR “electrode acupuncture” [TIAB] OR “electronic acupuncture” [TIAB]
#3 : “acupotomy” [TIAB] OR “acupotome” [TIAB] OR “needle knife” [TIAB] OR “acupotomology” [TIAB] OR “miniscalpel acupuncture” [TIAB] OR “miniscalpel needle” [TIAB] OR “stiletto needle” [TIAB] OR “xiaozhendao” [TIAB] OR “needle scalpel” [TIAB] OR “sword like needle” [TIAB]
#4 : “pharmacopuncture” [TIAB] OR “herb acupuncture” [TIAB] OR “herbal acupuncture” [TIAB] OR “herbalized acupuncture” [TIAB] OR “pharmaco-acupuncture” [TIAB] OR “pharmaco-puncture” [TIAB] OR “pharmacoacupuncture” [TIAB] OR “pharmacopuncture therapy” [TIAB]
#5 : #1 AND (#2 OR #3 OR #4)

1.2. Embase

('echography'/exp OR 'diagnostic ultrasonic examination':ti,ab,kw OR 'diagnostic ultrasonic imaging':ti,ab,kw OR 'diagnostic ultrasonic method':ti,ab,kw OR 'diagnostic ultrasound':ti,ab,kw OR 'doptone':ti,ab,kw OR 'duplex echography':ti,ab,kw OR 'echogram':ti,ab,kw OR 'echographic evaluation':ti,ab,kw OR 'echography':ti,ab,kw OR 'echoscopy':ti,ab,kw OR 'echosound':ti,ab,kw OR 'high resolution echography':ti,ab,kw OR 'scanning, ultrasonic':ti,ab,kw OR

'sonogram':ti,ab,kw OR 'sonographic examination':ti,ab,kw OR 'sonographic screening':ti,ab,kw OR 'sonography':ti,ab,kw OR 'ultrasonic detection':ti,ab,kw OR 'ultrasonic diagnosis':ti,ab,kw OR 'ultrasonic echo':ti,ab,kw OR 'ultrasonic examination':ti,ab,kw OR 'ultrasonic scanning':ti,ab,kw OR 'ultrasonic scintillation':ti,ab,kw OR 'ultrasonogram':ti,ab,kw OR 'ultrasonographic examination':ti,ab,kw OR 'ultrasonographic screening':ti,ab,kw OR 'ultrasonography':ti,ab,kw OR 'ultrasound diagnosis':ti,ab,kw OR 'ultrasound scanning':ti,ab,kw OR 'ultrasound'/exp OR 'phonophoresis':ti,ab,kw OR 'radiation, ultrasonic':ti,ab,kw OR 'sonication':ti,ab,kw OR 'sonification':ti,ab,kw OR 'ultra sound':ti,ab,kw OR 'ultrashell':ti,ab,kw OR 'ultrasonic':ti,ab,kw OR 'ultrasonic energy':ti,ab,kw OR 'ultrasonic irradiation':ti,ab,kw OR 'ultrasonic measurement':ti,ab,kw OR 'ultrasonic sound':ti,ab,kw OR 'ultrasonic wave':ti,ab,kw OR 'ultrasonic waves':ti,ab,kw OR 'ultrasonics':ti,ab,kw OR 'ultrasound':ti,ab,kw OR 'ultrasound radiation':ti,ab,kw OR 'ultrasound guided':ti,ab) AND ('acupuncture'/exp OR 'acupuncture':ti,ab,kw OR 'acupuncture therapy':ti,ab,kw OR 'shonishin':ti,ab,kw OR 'dry needling'/exp OR 'dry needle technique':ti,ab,kw OR 'dry needle therapy':ti,ab,kw OR 'dry needling':ti,ab,kw OR 'intramuscular stimulation':ti,ab,kw OR 'muscle dry needling':ti,ab,kw OR 'myofascial trigger point dry needling':ti,ab,kw OR 'trigger point dry needling':ti,ab,kw OR 'needling'/exp OR 'electroacupuncture'/exp OR 'acupuncture, electric':ti,ab,kw OR 'electric acupuncture':ti,ab,kw OR 'electrical acupoint stimulation':ti,ab,kw OR 'electrical acupuncture':ti,ab,kw OR 'electro-acupuncture':ti,ab,kw OR 'electroacupuncture':ti,ab,kw OR 'electrode acupuncture':ti,ab,kw OR 'electronic acupuncture':ti,ab,kw OR 'acupotomy'/exp OR acupotome:ti,ab OR 'needle knife'/exp OR acupotomology:ti,ab OR 'miniscalpel acupuncture':ti,ab OR 'miniscalpel needle':ti,ab OR 'stiletto needle':ti,ab OR xiaozhendao:ti,ab OR 'needle scalpel':ti,ab OR 'sword like needle':ti,ab OR 'pharmacopuncture'/exp OR 'herb acupuncture':ti,ab,kw OR 'herbal acupuncture':ti,ab,kw OR 'herbalized acupuncture':ti,ab,kw OR 'pharmaco-acupuncture':ti,ab,kw OR 'pharmaco-puncture':ti,ab,kw OR 'pharmacoacupuncture':ti,ab,kw OR 'pharmacopuncture':ti,ab,kw OR 'pharmacopuncture therapy':ti,ab)

1.3. cochrane Library

#1 : MeSH descriptor: [Acupuncture] explode all trees

#2 : MeSH descriptor: [Ultrasonography] explode all trees

#3 : MeSH descriptor: [Ultrasonics] explode all trees

#4 : Ultrasonography OR echography OR diagnostic ultrasonic imaging OR diagnostic ultrasound OR doptone OR duplex echography OR echogram OR echographic evaluation OR echography OR echoscopy OR echosound OR high resolution echography OR sonogram OR sonographic examination OR sonographic screening OR sonography OR ultrasonic detection OR ultrasonic diagnosis OR ultrasonic echo OR ultrasonic examination OR ultrasonic scanning OR ultrasonogram OR ultrasonographic examination OR ultrasonographic screening OR ultrasonography OR ultrasound diagnosis OR ultrasound scanning OR ultrasound OR phonophoresis OR radiation OR ultrasonic OR sonication OR sonification OR ultra sound OR ultrasonic OR ultrasonic energy OR ultrasonic irradiation OR ultrasonic measurement OR ultrasonic sound OR ultrasonic wave OR ultrasonic waves OR ultrasonics OR

ultrasound OR ultrasound radiation OR ultrasound-guided
#5 : acupuncture OR acupuncture therapy OR shonishin OR dry needling OR dry needle technique OR dry needle therapy OR intramuscular stimulation OR muscle dry needling OR myofascial trigger point dry needling OR trigger point dry needling OR needling OR electroacupuncture OR electric acupuncture OR electrical acupoint stimulation OR electrical acupuncture OR electro-acupuncture OR electrode acupuncture OR electronic acupuncture
#6 : acupotomy OR acupotome OR needle knife OR acupotomology OR miniscalpel acupuncture OR miniscalpel needle OR stiletto needle OR xiaozhendao OR needle scalpel OR sword like needle
#7 : pharmacopuncture OR herb acupuncture OR herbal acupuncture OR herbalized acupuncture OR pharmaco-acupuncture OR pharmaco-puncture OR pharmacoacupuncture OR pharmacopuncture therapy
#8 : (#2 OR #3 OR #4) AND (#1 OR #5 OR #6 OR #7)
