# Supplementary material for: Analysis of Research Trends in Ultrasound-Guided Acupuncture and Dry-Needling: A Scoping Review
Source: J Clin Med. 2024 Aug 22;13(16):4962. doi: 10.3390/jcm13164962 (PMC11355774; doi:10.3390/jcm13164962)
Supplement: Supplementary file 1 [file jcm-13-04962-s001.zip › Appendix S3. Disease Categories Based on ICD-11 Classifications.pdf]

S3 Appendix. Disease Categories Based on ICD-11 Classifications

Codes of ICD-11	DISEASE CATEGORIES	N(%)
01 Certain infectious or parasitic diseases	1E91.5 Postherpetic polyneuropathy	1 (1.2%)
02 Neoplasms	2B91.Z Malignant neoplasms of rectosigmoid junction, unspecified	1 (1.2%)
05 Endocrine, nutritional or metabolic diseases	5B81 Obesity	1 (1.2%)
08 Diseases of the nervous system	8B82.0 Trigeminal neuralgia	2 (2.3%)
	8B93.8 Radiculopathy due to spondylosis	1 (1.2%)
	8C10.0 Carpal tunnel syndrome	1 (1.2%)
	8C11.00 Sciatic nerve piriformis syndrome	4 (4.7%)
	8C11.3 Lesion of common peroneal nerve	1 (1.2%)
	8C1Z Mononeuropathy of unspecified site & 8C11.2 Lesion of femoral nerve	1 (1.2%)
10 Diseases of the ear or mastoid process	AB32.0 Persistent Postural-Perceptual Dizziness	1 (1.2%)
12 Diseases of the respiratory system	CA08.3 Vasomotor rhinitis	1 (1.2%)
	CB21 Pneumothorax	1 (1.2%)
	CB23 Disorders of diaphragm	1 (1.2%)
15 Diseases of the musculoskeletal system or connective tissue	FA01 Osteoarthritis of knee	6 (7.0%)
	FA31.3 Acquired wrist drop	1 (1.2%)
	FA5Y Other specified arthropathies & XA05J7 Shoulder joint	1 (1.2%)
	FA80.1 Intervertebral disc degeneration of cervical spine with prolapsed disc	1 (1.2%)
	FA80.9 Intervertebral disc degeneration of lumbar spine with prolapsed disc	4 (4.7%)
	FA8Z Degenerative condition of spine, unspecified	2 (2.3%)
	FA91 Infection of intervertebral disc	1 (1.2%)
	FB32.Y Other specified disorders of muscles	1 (1.2%)
	FB32.4 Contracture of muscle	1 (1.2%)
	FB40.1 Plantar fasciitis	4 (4.7%)

	FB40.3 Calcific tendinitis	2 (2.3%)
	FB40.4 Trigger finger	3 (3.5%)
	FB40.5 Radial styloid tenosynovitis	2 (2.3%)
	FB40.Y Other specified tenosynovitis	3 (3.5%)
	FB53.0 Adhesive capsulitis of shoulder	4 (4.7%)
	FB53.1 Rotator cuff syndrome	1 (1.2%)
	FB55.1 Lateral epicondylitis of elbow	4 (4.7%)
	FB55.Z Enthesopathies, unspecified	4 (4.7%)
	ME84.2 Low back pain	3 (3.5%)
16 Diseases of the genitourinary system	GB70.1 Calculus of ureter	1 (1.2%)
21 Symptoms, signs or clinical findings, not elsewhere classified	MD93 Dysphagia	1 (1.2%)
	MB47.3 Cramp of spasm	1 (1.2%)
	MG30.01 Chronic widespread pain	10 (11.6%)
	MG30.02 Chronic primary musculoskeletal pain	3 (3.5%)
	MG30.21 Chronic postsurgical pain	1 (1.2%)
	MG30.04 Complex regional pain syndrome	1 (1.2%)
22 Injury, poisoning or certain other consequences of external causes	ND51.2 Injury of spinal cord, level unspecified	1 (1.2%)
26 Supplementary Chapter Traditional Medicine(TM) Conditions - Module I	SC62 Numbness disorder (TM1)	1 (1.2%)
V Supplementary section for functioning assessment	VV12 Sensation of pain	1 (1.2%)
Total		86 (100%)

ICD : International Classification of Diseases.
