# Supplementary material for: Analysis of Research Trends in Ultrasound-Guided Acupuncture and Dry-Needling: A Scoping Review
Source: J Clin Med. 2024 Aug 22;13(16):4962. doi: 10.3390/jcm13164962 (PMC11355774; doi:10.3390/jcm13164962)
Supplement: Supplementary file 1 [file jcm-13-04962-s001.zip › Appendix S4. The information of included Systematic Review and Meta-analysis.pdf]

S4 Appendix. The information of included Systematic Review and Meta-analysis

Study ID	Study type	Included studies	Participants	Intervention method	Comparison	Target disease	Main results
Shomal Zadeh F (2023) [15]	Systematic Review	RCT (12 studies)	n=481	PUNT	alternative treatments (surgical tenotomy, →, SI)	Chronic tendinopathy	Although PUNT is an effective treatment technique for chronic tendinopathy, the effects of PRP and SI are temporary and do not contribute to long-term results.
Lin S (2023) [16]	Meta-analysis	RCT (NA)	n=543	Ultrasound-guided acupotomy	NA	Knee osteoarthritis (KOA)	UGAT was significantly more efficient than the control in reducing VAS scores (mean difference = -0.81, 95% confidence interval (CI) = [-1.15, -0.47], p < 0.00001, 8 studies), improved knee function in the Lysholm Knee Score (mean difference = 8.26, 95% CI = [1.56, 14.97], p = .02, 2 studies), and increased clinical efficacy rate (relative risk = 1.14, 95%). CI) = [1.06, 1.23], p = .0005, 6 studies).
Shomal Zadeh F (2023) [17]	Meta-analysis	29 studies	n=1674(1876 tendons)	PUNT	NA	chronic tendinopathy, fasciopathy	PUNT significantly alleviated pain with the standard mean difference of 2.5 (95% CI: 2.0-3.0; p < 0.05), 2.2 (95% confidence interval (CI): 1.8-2.7; p < 0.05), and 3.6 (95% CI: 2.8-4.5; p < 0.05) points in short-term, intermediate-term, and long-term follow-up intervals, respectively
Liang YS (2023) [18]	Meta-analysis	RCT (15 studies)	n=988	Ultrasound-guided acupotomy	traditional acupotomy, traditional operation or injection of medication	Trigger finger	Meta-analysis showed that the overall clinical effectiveness (OR = 4.83; 95% CI 2.49-9.37; I2 = 73.1%; P < 0.001) in the experimental group was significantly better than that of the control group
Notes. PUNT: percutaneous ultrasound-guided needle tenotomy; PRP: platelet-rich plasma injection; SI: steroid injection; UGAT: ultrasound-guided acupotomy;							
