# Supplementary material for: Analysis of Research Trends in Ultrasound-Guided Acupuncture and Dry-Needling: A Scoping Review
Source: J Clin Med. 2024 Aug 22;13(16):4962. doi: 10.3390/jcm13164962 (PMC11355774; doi:10.3390/jcm13164962)
Supplement: Supplementary file 1 [file jcm-13-04962-s001.zip › Appendix S5. The information of included Clinical Trial.pdf]

S5 Appendix. The information of included Clinical Trial

Study ID	Study type	Objective	Participants (Male, Female)	Targetting area	Intervention methods	Main results
Sun W (2015) [86]	non-comparative study	To investigate the relationship between catgut embedding therapy needling sensation and implantation depth for improved safety	20(NA)	EX-B2	Needle insertion under the ultrasound	Implanting catgut in various layers resulted in different sensations, with the most significant distending pain in the myofascial layer. Qi arrival was observed in all layers, and a 2.5 cm length was commonly implanted deeply into the multifidus, lasting an average of 72.0 ± 10.2 hours.
Ding Y (2012) [99]	non-comparative study	To evaluate the feasibility and safety of visualized acupotomy.	52(NA)	Treatment at appropriate lesion site after ultrasound detection (cervical spine, lumbar spine, etc.)	ultrasound-guided acupotomy	VAS score: 6.560 +/- 0.893 → 1.058 +/- 0.857 ($p < 0.05$).<br>Curative rate: 48.1% (25/52) (after one treatment)<br>Total effective rate: 98.1% (51/52).<br>HSS and CMS were improved (both $p < 0.05$).
Liu J (2021) [88]	non-comparative study	To investigate the ultrasonic anatomical characteristics and needle-knife insertion approach in KOA	60(14/46)	"Hedingci" "Binwaixia" "Binneixia" and "Yinlingshang" /	needle-knife stimuation	"Hedingci" lesions : mainly located at the attachment of quadriceps tendon to patella and suprapatellar bursa<br>"Binwaixia" and "Binneixia" : mainly located at the attachment of retinaculum patellae laterale and retinaculum patellae mediale to patella and infrapatellar fat pad<br>"Yinlingshang" : mainly located at the attachment of goose foot tendon to medial tibial condyle and bursa of goose foot
Park JJ (2011) [87]	non-comparative study	To examine the frequency of sensations during US-guided needling to specific tissue levels	5(3/2)	LU4, LI13	ultrasound guided acupuncture needling	Pricking, sharp sensations : shallower TLs > deeper ($p=0.007$)<br>Deep, dull, heavy, spreading, electric shocks sensations : deeper TLs > shallower TLs ($p=0.002$)
Chen X (2021) [89]	non-comparative study	Simultaneously comparing bioelectric signals from different acupuncture points to analyze their types and assist in assessing the patient's condition	NA	Shang ju xu / Zu san li	Acupuncture+ multistage electrode probe	Acupoints exhibit high activity levels, indicating feasibility of collecting electric signals around them in this hardware system
Bubnov R (2019) [100]	non-comparative study	To evaluate efficacy of dry needling under US guidance for treatment myofascial and neuropathic components of LBP	52(15/37)	MTrP under US guidance	Needling using ultrasound guidance	VAS scores: 7.4 → 2.2<br>LANSS scores: 16 → 4<br>US shows improved nerve structure and increased muscle contractility during functional tests and walking across all

						levels
Leow MQH (2017) [93]	non-comparative study	To analyze real-time changes in blood vessel size, flow velocity, muscle strain before and after acupuncture needle insertion and removal	2(1/1)	Transversely approximately 1 cm proximal to the cubital crease, over the biceps muscle. Needle insertion LU5(Chize)	acupuncture using ultrasound	Blood flow rate decreased after needling, but subsequent changes were subjective. Prior exposure to acupuncture may affect the effectiveness of needling.
Maher RM (2013) [101]	non-comparative study	To assess if dry needling reduces shear modulus in upper trapezius muscle MTrPs acutely and if posture change affects shear modulus	7(0/7)	the trigger point in the upper trapezius muscle	Dry needling	After dry needling and transitioning to prone position, stiffness decreased noticeably. Shear modulus significantly reduced post-DN ($p < 0.01$) and from sitting to prone ($p < 0.05$).
Litscher G (1998) [94]	non-comparative study	To investigate cerebral effects during acupuncture on healthy volunteers using the new TCD-three-dimensional imaging method.	12(8/4)	PC6, CV6, ST36, SP6	Acupuncture	Significant ($p < 0.01$; ANOVA; Tukey test) increase in mean blood flow velocity, which was measured at different depths of the right middle cerebral artery.
Langevin HM (2004) [95]	non-comparative study	To quantify tissue displacement during acupuncture manipulation in humans.	12(NA)	Two distal and two proximal points bilaterally, located 18 cm (A) and 12 cm (B) proximal to the middle of the superior edge of the patella	Acupuncture using ultrasound elastography techniques	Tissue displacement were noted between upward needle motions 1 and 2 (1 > 2) without rotation. A similar trend was observed in rebound tissue displacement
Streitberger K (2007) [90]	non-comparative study	To investigate the distance between the needle tip and the median nerve during acupuncture at P6.	50(11/39) / Total 77 cases	P6	Dry needling and ultrasound imaging	The mean distance from the needle tip to the nerve was 1.8 mm (standard deviation 2.2; range 0–11.3). No association between the number of nerve contacts and de qi.
Tang CT (2019) [91]	non-comparative study	To describe the effects of acupuncture needling to the radial nerve in a single healthy subject	1(1/0)	LI13	Dry needling and ultrasound imaging	The radial nerve was seen to “roll” out of the way when it was needled. Two De Qi responses were elicited en route to the nerve, but, during nerve contact and penetration, minimal-to-no sensation was experienced.
Leow MQ (2016) [96]	non-comparative stud	To evaluateits feasibility and to discuss theimplications for education and research.	1(NA)	LU11, LU10, LU7	Dry needling and ultrasound imaging	Ultrasound showed the needle clearly in both longitudinal and transverse planes, allowing observation of its position relative to tissue and bone. The needle tip appeared as a small white spot at acupuncture points.

	y					
Leow MQH (2017) [97]	non-comparative study	To explore the potential use of ultrasound in locating the second posterior sacral foramen acupuncture point	1(NA)	the second posterior sacral foramen acupuncture point	Ultrasound-guided technique for needle insertion in acupuncture practice	The needle was inserted to a depth of 4.0 cm to reach the surface of the sacral foramen.
Yang J (2022) [102]	non-comparative study	To evaluate ultrasound-guided A1 pulley release by acupotomy on cadavers for safety and effectiveness.	6 Cadavers (3/3)	A1 Pulley	Ultrasound-Guided Acupotomy Ultrasound Guided 21-gauge needle)	Compared with the needle, ultrasound-guided acupotomy release is safer ($p < 0.05$).<br><br>The ultrasound-guided acupotomy technique achieved a complete A1 pulley release more frequently than the needle technique (93.3% vs. 36.7%, $p < 0.05$).
Zhu X (2019) [98]	non-comparative study	To investigate percutaneous release procedures in the lumbar region under ultrasound guidance using acupotomy	12 Cadavers (8/4)	LF in L3/L4, L4/L5, and L5/S1	Ultrasound-Guided Percutaneous Release Procedures in the LF by Acupotomy	The measured data for the transverse-axis approach for L3/L4, L4/L5, and L5/S1 segments showed that there were no differences in the needle angle, the depth of needle penetration, and the distance from the spinous process to the puncturepoint
Chen HN (2018) [92]	non-comparative study	To assess the depth of the GB 21 acupoint in adults and its relevance for safe acupuncture practices	101(41/60)	GB21	Ultrasonography	The depth of GB 21 increased with body weight, height, and BMI ($p < 0.001$). The mean depth of GB 21 in men (17.4 mm) was greater than that in women (14.6 mm; $p < 0.001$). The depth difference between the right and left shoulders was significant in men ($p < 0.001$) but not in women.
Gascon-Garcia J (2018) [103]	non-comparative study	To use ultrasound imaging to show how the needles in dry needling applied in the carpal tunnel can reach the transverse carpal ligament	18(8/10)	carpal tunnel	Dry needling with the fascial winding technique in the carpal tunnel using ultrasound	93.1% of the needles placed came into contact with the transverse carpal ligament with traction-stretching of the ligament observed when the needles were manipulated with the fascial winding technique in 80.6%.
Wang-Price SS (2022) [104]	non-comparative study	To determine the validity and reliability of needle placement of two DN protocols for the LM in individuals with a high BMI.	20(8/12)	lumbar multifidus	Dry needling and ultrasound imaging	The probability of reaching the deep LM muscle was high (85–95%) at L4 and L5
Shen Y (2021) [105]	non-comparative stud	To observe (1) the feasibility of ultrasonographic evaluation of safe needling depth before acupuncture (2) the safe depth at abdominal acupuncture points.	20 Cadavers (15/5)	CV12, 9, 6, 4 / ST21, 25, 28, / SP15 / GB26	Dry needling	On average, the maximum depth (subcutaneous to peritoneal) at the site of abdominal acupuncture points was 1.27 ± 0.96 (0.29–3.21) cm, the safe depth was 0.89 ± 0.67 (0.2–2.25) cm, the fat thickness was 0.92 ± 0.76 (0.12, 2.43) cm and the muscle thickness was 0.43 ± 0.4 (0.09–2.04) cm

	y					
Fernández-de-Las-Peñas C (2017) [106]	non-comparative study	To determine if a needle is able to reach the cervical multifidus during the application of dry needling or acupuncture.	5 Cadavers (3/2) + 2 people (2/0)	1cm lateral to the spinous process at C3-C4. The needle was advanced from a posterior to anterior direction into the cervical multifidus with a slight inferior medial angle (approximately 10° to reach the vertebra lamina	Dry needling and ultrasound imaging	The needle does pierce the cervical multifidus muscle during insertion and that the tip of the needle rests properly against the vertebral laminae, thereby guarding the sensitive underlying spinal structures from damage.
Hemani JV (2023) [107]	non-comparative study	To demonstrate that TGN pain stems from both neural activity and end plate abnormalities, including end plate noise, ATP deficiency, and myofascial trigger points in affected muscles.	10(NA)	Terminal Branches of Trigeminal Nerve	Trigeminal Neuralgia (TGN)	The difference in all the parameters of pain before and after USGDN was statistically significant.
Yeo A (2016) [108]	non-comparative study	To target both pathological sites by combining dry needling with percutaneous hydrostatic decompression as a novel treatment.	21(NA)	Achilies tendon	Chronic Achilles tendinopathy	Twenty-four tendons (in 19 patients) were successfully treated.
Fernández-de-Las-Peñas C (2020) [109]	non-comparative study	To examine if a solid needle is able to properly penetrate the obliquus capitis inferior muscle during the application of dry needling	15(10 pain-free healthy subjects and 5 fresh cadavers)	obliquus capitis inferior muscle, C2 laminae	Dry needling	Both the cadaveric and ultrasonic studies showed that the needle penetrated the obliquus capitis inferior muscle during its insertion and that the tip of the needle rested against C2 laminae, thereby reaching the targeted muscle.
Notes. LBP, Low back pain; LANSS, leeds assessment of neuropathic symptoms and signs; TCD, transcranial doppler sonography; LF, lumbar ligamentum flavum; LM, lumbar multifidus; USGDN : ultrasound-guided dry needling.						
