# Supplementary material for: Analysis of Research Trends in Ultrasound-Guided Acupuncture and Dry-Needling: A Scoping Review
Source: J Clin Med. 2024 Aug 22;13(16):4962. doi: 10.3390/jcm13164962 (PMC11355774; doi:10.3390/jcm13164962)
Supplement: Supplementary file 1 [file jcm-13-04962-s001.zip › Appendix S6. The Information of included cohort study.pdf]

S6 Appendix. The Information of included cohort study

Study ID	Study type	Intervention method	Participants	Target disease	Outcome	Main result
James SL (2007)<br><br>[110]	Prospective cohort study	Dry needling (US guided) and autologous blood injection	44(47 knees in 44 patients')	patellar tendinosis	Pre- and post-procedure VISA	Pre-procedure mean score 39.8 points (range 8–72 points) vs. post-procedure mean score 74.3 points (range 29-100 points), p<0.001<br><br>mean follow-up period 14.8 months (range 6-22 months): with therapeutic intervention.<br><br>VISA scores improved
Hameed M (2016)<br><br>[111]	Prospective cohort study	Ultrasound-guided dry needling and percutaneous high volume stripping	64	Achilles tendinopathy	Pain score, Patient's satisfaction	54 out of 64 tendons were successfully treated<br><br>improved in pain scores and the majority of the patients (>85%) are satisfied.
