# Supplementary material for: Analysis of Research Trends in Ultrasound-Guided Acupuncture and Dry-Needling: A Scoping Review
Source: J Clin Med. 2024 Aug 22;13(16):4962. doi: 10.3390/jcm13164962 (PMC11355774; doi:10.3390/jcm13164962)
Supplement: Supplementary file 1 [file jcm-13-04962-s001.zip › Appendix S7. The Information of included RCT and nRCT.pdf]

S7 Appendix. The Information of included RCT and nRCT

Study ID	Study type	Participant count (intervention/control)	Intervention	control	intervention type	target disease	treatment point	outcome	Main result
Ding Y (2013) [33]	RCT	n=80 (40/40) (Twenty people were distributed for each disease.)	A : Interventional ultrasound combined with acupotomy	B : conventional acupotomy	Ultrasound-guided acupotomy with lidocain	Shoulder joint disorder	Treatment based on ultrasound findings	VAS, CMC-Murley Shoulder function evaluation scale	A: 51.85 ± 9.56 → 91.25 ± 5.75, t value 11.17 ($p < 0.01$)<br>B: 52.28 ± 7.96 → 75.72 ± 8.56, t value 6.34 ($p < 0.01$)
						Knee osteoarthritis	Treatment based on ultrasound findings	VAS, HSS scale	A: 42.70 ± 9.19 → 90.40 ± 7.35 t value 12.820 ($p < 0.01$)<br>B: 43.23 ± 7.56 → 75.54 ± 9.21 t value 8.575 ($p < 0.01$)
						Lumbar disc herniation	spinous process space / superior and inferior articular processes	VAS, M-JOA scoring table	No statistically significant effect on the results between A and B (both $p>0.05$).
						Cervical disc herniation	spinous process space / superior and inferior articular processes	VAS, Cervical Function Assessment Form	
Duan H (2016) [34]	RCT	n=234, 117(male 54, female 63)/117	A : Ultrasound-guided acupotomy group	B : Traditional knife group	Ultrasound-guided acupotomy with lidocain	Plantar fasciitis (16.98 ± 8.99 months)	A : lesion detected by ultrasound<br>B : The point detected by operator with the thumb	VAS	Immediately after treatment: A ≒ B<br>After 1 and 3 months –<br>A: 6.55 ± 1.69 → 0.8 ± 0.16, B: 6.55 ± 1.52 → 1.48 ± 0.13.
								Tenderness score	
								AOFAS-AH score	A significantly improved compared to B
Chen T (2021) [49]	RCT	n=70 (35,5 cases dropped off/(35,4 cases dropped off)	A : Shallow-tissue thread embedding group	B: Deep-tissue thread embedding group	Ultrasound-guided acupoint thread embedding therapy	Obesity	CV3, CV9, CV10, CV12, SP15, ST25, ST29	Body mass, BMI, waist circumference, hip circumferenc	BMI and waist circumference: A > B ($p < 0.05$)<br>Distention and fullness sensation and needling sensation and intensity: A > B ($p < 0.05$).

								e	
Zhang WB (2019)<br>[43]	RCT	n=74 (37/37)	A : Treated with ultrasound-guided intrathecal injection + Releasing method of needle-knife	B : Treated with ultrasound-guided intrathecal injection	Ultrasound-guided acupotomy+Intrathecal injection	Stenosing tenosynovitis of flexor tendon (trigger finger)	Ashi points	Self-made 9-score scale	Excellent/good rate: A > B ($p < 0.05$)<br>Cure rate:<br>A: 100.0% → 97.3%<br>B: 13.5% → 10.8%
Lan X (2023)<br>[19]	RCT	n=72 (42/30)	A : SNK group	B : OS group	Ultrasound-guided Acupotomy	Patients with grade 2 and above trigger digits	NA	VAS, QG	VAS, QG of both groups decreased significantly. (No significant difference)
Cao XY (2019)<br>[44]	RCT	n=36 (18/18)	A : ESWT+USGAP	B : ESWT Group	ESWT+Ultrasound-guided acupotomy	Frozen shoulder	shoulder's Ashi point	NRS	A: 6.5 → 2.7<br>B: 6.7 → 4.3<br>A < B ($p < 0.01$).
								SPADI	A: 54.9 → 30.4<br>B: 56.1 → 43.2<br>A < B ($p < 0.01$).
Bubnov RV (2013)<br>[35]	RCT	n=133 (91/42)	A : US guidance group	B : Conventional dry needling group (without US guidance)	Ultrasound-guided dry needling	Myofascial Pain	Trigger point	VAS	A: 7.2 → 1.1 (pain reduced by 84%) ($p < 0.001$)<br>B: 7.4 → 2.7 (pain reduced by 63.5%) ($p < 0.001$)
								Number of needles used in treatment	A: 2.6–0.54<br>B: 4.45–0.7
								Number of times a muscle twitch response was induced	A: 92.26–3.8%<br>B: 58.8–7.5%
								Number of TrP session	A: 2.3<br>B: 1.7–3.6
Bubnov R (2019)<br>[20]	RCT	n=40 (12 females)	A : Dry needling under ultrasound guidance Group	B : High-Energy Extracorporeal Shockwave Therapy Group	Ultrasound-guided dry needling	Chronic low back pain	MTrP	VAS	A: 7.4 → 2.3 ($p < 0.05$).<br>Structure: improved<br>Mobility, contractility: increased<br>B: 7.2 → 5.2, but then recurred.

Liu JY (2019) [36]	RCT	n=52(26 – 3 eliminated / 26)	A : Ultrasound-guided acupoint electrical stimulation group	B : Conventional acupoint electrical stimulation group	Ultrasound-guided acupoint electrical stimulation	Ventilator-induced diaphragmatic dysfunction	Zhangmen (LR 3), Dabao (SP 21), Pishu (BL 20), Shenshu (BL 23)	Mechanical ventilation time	A < B (p < 0.05).
								intensive care unit time	A ≒ B (p > 0.05).
								total hospitalizatio n time	A ≒ B (p > 0.05).
								hospital mortality rate	A ≒ B (p > 0.05).
								reintubation rate	A < B (p < 0.05).
Ding Y (2016) [21]	RCT	n=60(NA/NA)	A : Ultrasound-guided acupotomy group	B : electro-acupuncture (EA) group	Ultrasound-guided acupotomy	knee osteoarthritis (KOA)	NA	ADL	ADL: A > B (p < 0.01).
								HSS	A: 58 ± 5 → 86 ± 5<br>B: 60 ± 4 → 77 ± 6<br>A > B (p < 0.01).
								VAS	A: 5.1 ± 1.1 → 2.1 ± 1.7<br>B: 5.1 ± 1.5 → 3.1 ± 1.2<br>A < B (p < 0.01)
								Degree of infrared thermal images	A: 0.81 ± 0.21 → 0.32 ± 0.12<br>B: 0.78 ± 0.25 → 0.33 ± 0.14 A ≒ B (p < 0.01).
Sun W (2015) [50]	RCT	n=90(30/30/30)	A: Deep-layer embedding group (multifidus muscle layer)	B : Middle-layer embedding group (semispinalis capitis muscle layer)<br>C : Shallow-layer embedding group	Ultrasound-guided embedding therapy	Cervical spondylosis	Jiaji (EX-B 2) and Dazhui (GV 14) in C5 and C6 on the affected side.	Symptoms and functional score	Significantly increased in A (p<0.05; compared to B, C. Both p < 0.05).
								PRI,VAS,PPI	Significantly decreased after treatment in A and B compared to C

				(subcutaneous layer),					(all p < 0.05).
								NDI	A and B decreased after treatment (p < 0.05), A decreased significantly compared to B,C (all p < 0.05).
Sharif F (2023) [45]	RCT	n=96(48/48)	A : UG-DN+CPT (Conventional physical therapy) Group	B : CPT Group	Ultrasound-guided dry needling + CPT	Jumper's knee	NA	VAS	A: 8 ± .00 → 3 ± 1<br>B: 8 ± 2 → 1.5 ± 1<br>A > B (p = 0.000).
								VISA-P questionnaire	A: 52 ± 8 → 83.5 ± 7<br>B: 51 ± 12 → 92 ± 2<br>A < B (p = 0.000).
								Lysholm scale	A: 67 ± 3 → 84 ± 5<br>B: 65 ± 8 → 92 ± 4<br>A < B (p = 0.000).
								KOOS	A: 52.5 ± 8 → 83.5 ± 8<br>B: 52 ± 12 → 92 ± 3<br>A < B (p = 0.000).
Benito-de-Pedro AI (2023) [22]	RCT	n=52(26/26)	A : Deep dry needling (DDN) group	B : Percutaneous electrolysis (PE) group	Ultrasound-guided dry needling	Active Myofascial Trigger Points of the Levator Scapulae	Levator scapulae	Pain intensity	A: 6.80 ± 1.13 → 3.00 ± 2.04<br>B: 6.77 ± 1.03 → 2.77 ± 2.29<br>A ≒ B (p > 0.05)
								PPT (at central MTrP)	A: 2.52 ± 0.70 → 2.74 ± 0.81<br>B: 2.56 ± 0.75 → 2.72 ± 0.77<br>A ≒ B (p > 0.05).
								Cervical ROM	A: 68.79 ± 8.50 → 74.13 ± 4.97<br>B: 72.06 ± 4.04 → 75.13 ± 3.84<br>A < B (p < 0.05).
								neck disability	A < B (p = 0.047).
								Post-needling soreness	A ≒ B (p > 0.05).
Sharif F (2022)	RCT	n=94(47/47)	A : Ultrasound-guided dry needling + CPT	B : CPT group	Ultrasound-guided dry needling + Conventional	Jumper's knee	NA	VAS	A: 8.20 ± 0.75 → 3.00 ± 1.43<br>B: 8.00 ± 0.64 → 6.40 ± 0.49<br>A < B ((p = 0.000).

[46]					physical therapy			VISA-P	A: 43.10 ± 7.08 → 88.70 ± 8.59<br>B: 40.70 ± 6.61 → 78.20 ± 8.34<br>A > B ($p = 0.000$).
								Lysholm scale	A: 56.00 ± 4.05 → 83.40 ± 5.78<br>B: 55.20 ± 2.16 → 80.20 ± 4.54<br>A > B ($p = 0.000$).
								KOOS	A: 56.56 ± 7.12 → 89.29 ± 5.19<br>B: 51.29 ± 7.6 → 79.40 ± 6.19<br>A > B ($p = 0.000$).
Qin X (2022)<br><br>[23]	RCT	n=68(34/34)	A : Ultrasound-guided 18G-PTC puncture needle group	B : Small needle-knife therapy group	Ultrasound-guided small needlg-knife	Primary frozen shoulder	NA	Overall efficacy	A : 88.23%<br>B : 67.64%<br>A > B ($p < 0.05$)
								UCLA scores of the shoulder joint	A : 13.61 ± 3.77 → 31.22 ± 3.34<br>B : 14.34 ± 3.89 → 25.43 ± 3.83<br>A > B ($p < 0.05$)
								shoulder mobility	The efficacy of the A was better compared to B ($p < 0.05$)
								muscle elasticity and thickness	The efficacy of the A was better compared to B ($p < 0.05$)
								VAS	Before treatment<br>A: 7.12 ± 1.44 B: 7.21 ± 1.45<br>($p > 0.05$)<br>After treatment<br>A: 3.41 ± 1.39, B: 5.02 ± 1.76<br>($p < 0.05$)
Shen Y (2022)<br><br>[37]	RCT	n=59(23/39)	A : Operated with US guidance	B : Operated without US guidance	Ultrasound-guided acupotomy	De Quervain's disease	NA	Regarding the amount of release	A: 20 cases (87%) vs B: 27 cases (75%)
Wang YH (2023)	RCT	n=60(NA/NA)	A : Received Juanbi Decoction 3 times daily for 2	B : Same protocol was used with the group A, but the	Ultrasound-guided Acupotomy + Juanbi tang	Lumbar disc herniation	NA	VAS, ,	A: 4.87 ± 0.57 → 3.67 ± 0.48<br>B: 4.8 ± 0.61 → 3.43 ± 0.73<br>A ≓ B ($p < 0.05$)

[51]			weeks along with an acupotomy assisted by ultrasound	Juanbi Decoction was replaced with normal saline				ODI	A: 66.20 ± 2.91 → 50.93 ± 5.79<br>B: 66.8 ± 4.80 → 47.4 ± 6.22 A > B ($p < 0.05$)
								LBOS	A: 24.57 ± 2.67 → 29.4 ± 3.94<br>B: 24.06 ± 2.24 → 30.67 ± 4.96<br>A ≒ B ($p < 0.05$)
								JOA	A: 11.23 ± 1.61 → 16.83 ± 2.60<br>B: 11.43 ± 1.65 → 18.67 ± 1.79<br>A < B ($p < 0.05$)
Zhang S (2019)<br>[47]	RCT	n=51(NA/NA)	A : steroid injection combined with US-guided MSN release group	B : steroid injection group	steroid administration alone / concurrent steroid administration using miniscalpel-needle	Carpal tunnel syndrome (CTS)	NA	BCTQ-SSS	A: 3.10 ± 0.32 → 1.84 ± 0.21 ($p = 0.096$)<br>B: 3.00 ± 0.25 → 2.06 ± 0.23 ($p < 0.001$)
								BCTQ-FSS	A: 3.10 ± 0.25 → 1.80 ± 0.35 ($p = 0.112$)<br>B: 3.00 ± 0.25 → 2.08 ± 0.27 ($p < 0.001$)
								CMAP	A: 9.4 ± 1.2 → 12.2 ± 1.3 ($p = 0.613$)<br>B: 9.5 ± 1.1 → 11.3 ± 1.1 ($p < 0.001$)
								DML	A: 5.2 ± 0.3 → 4.5 ± 0.4 ($p = 0.002$)<br>B: 5.4 ± 0.3 → 4.7 ± 0.4 ($p < 0.001$)
								SNAP	A: 12.1 ± 1.8 → 16.3 ± 3.5 ($p = 0.368$)<br>B: 12.0 ± 1.6 → 15.4 ± 2.7 ($p < 0.001$)
								SNCV	A: 38.6 ± 3.8 → 46.5 ± 2.5 ($p < 0.597$)<br>B: 39.5 ± 3.2 → 44.7 ± 3.2 ($p < 0.001$)
								CSA	A: 13.3 ± 1.4 → 10.8 ± 1.1 ($p = 0.493$)<br>B: 13.1 ± 1.5 → 11.6 ± 1.2 ($p < 0.001$)
Bureau NJ (2022)<br>[24]	RCT	n=62(NA/NA)	A : Dry needling (US guieded)	B : Surgery	Ultrasound-guided dry needling	Chronic lateral epicondylosis	Lateral epicondyle	PRTEE score	B 33.4 (CI 25.2 – 41.5) > A 26.9 (CI 19.4 – 34.4)<br>($p = 0.25$).

								Proportion of successful treatment	B 83% (CI 63 – 95%) > A 81% (CI 63 – 93%) (p = 1.00).
Bubnov R (2011) [38]	RCT	N=133(91/42)	A : Dry needling (US guieded)	B : Dry needling	Ultrasound-guided dry needling	MPS (myofascial pain syndrome)	Trigger points	pain relief effect, and level of inducing local twitch response (LTR)	Increased in A
								Average number of needling trigger points, Average number of treatment sessions	Decreased in A
Bubnov RV (2015) [52]	RCT	n=32(NA/NA)	A : Received dry needling (DN) of paravertebral ("central") MTrP under ultrasound guidance	B : Received DN under ultrasound guidance of "peripheral" MTrP in muscles	Ultrasound-guided dry needling	LBP (Low back pain)	Trigger points	VAS	A: 7.2 → 1.2<br>B: 7.3 → 3.5<br>A < B (p<0.05)
								PainDetects (1-38) scores	A: 98% (18.3 → 9.2)<br>B: 25% (18.5 → 11.5)<br>A > B (p < 0.01)

								MTrP recurrence	A: 25%, B: 58% ($p < 0.01$) at 24 hours after manipulation; outcome at 7th day was A: 7%, B: 35% ($p < 0.05$).
Samiei SM (2021) [25]	RCT	n=34(NA/NA)	A : Ultrasound-guided Dry needling with Mulligan mobilization technique (DN with MM)	B : Only dry needling (DN)<br>C : Don't received any intervention	Ultrasound-guided dry needling + Mulligan mobilization technique	lateral epicondylitis	extensor muscles	Pain intensity, Function level	All variables in A and B had a significant improvement compared to C.<br>Function and VAS scores : A was better than B
								Tendon Thickness of extensor muscles	A ≒ B
De Boer FA (2017) [26]	RCT	n=25(NA/NA)	A : Dry needling (US guieded)	B : Radial Shockwave (RSWT)	Ultrasound-guided dry needling	Shoulder Calcific tendinitis	NA	NRS	A : 7.5 → 1.9<br>B : 7.9 → 2.1<br>no significant differences were found
								Oxford	A: 38.5 → 53.2<br>B : 38.5 → 49.1<br>no significant differences were found
Zhu Ting (2018) [61]	RCT	n=52(26/26)	A : Drug injection and acupotomy (US guieded)	B : Drug injection and cupotomy (under the guidance of palpation)	Ultrasound-guided acupotomy + Drug injection	De Quervain disease	Abductor pollicis longus and extensor pollicis brevis tendons and tendon sheaths in styloid process of	VAS, Quinnell scoring	VAS scores, Quinnell scores of A decreased compared with those of B (all $p < 0.05$)

							radius		
Tabatabaiee A (2019) [62]	RCT	n=32(16/16)	A : Dry needling (US guieded) + Advice	B : Waitlist control group (Only advice)	Ultrasound-guided dry needling	Piriformis muscle syndrome (PMS)	Piriformis muscle	ODI, PPT, transverse-plane hip ROM	Pain intensity was decreased in A than in B (-2.16 [-1.01 to -3.32], (p = 0.007).
Xie N (2019) [27]	RCT	n=48(24/24)	A : ultrasound-guided dry needling for myofascial trigger points + with stretching training	B : sole non-weight-bearing plantar fascia stretching	Ultrasound-guided dry needling	Plantar fasciitis	Trigger points on gastrocnemius and soleus	NPRS, AOFAS, PCS, MCS, SF-36	The overall differences of NPRS, AOFAS, PCS and MCS were significant before and after treatment in both two groups (all p = 0.05).
Huang Y (2022) [28]	RCT	n=54(28/26)	A : Dry needling (US guieded)	B : pharmacotherapeutic group	Ultrasound-guided dry needling	Posttherpetic neuralgia mixed with myofascial pain syndrome	Trigger points	VAS, MPQ	Effective rate (VAS score <3 or MPQ socre <2): 92.9% vs 38.5% (A vs B) (p < 0.01)<br>Recurrent rate (VAS score >3 or MPQ score >2): 7.1% vs 34.6% (A vs B) (p = 0.02)<br>Satisfactory rate: A > B
Pang JCY (2022) [29]	RCT	n=84(28/28/28)	A : Dry needling (US guided) + exercise	B : Placebo US guided DN with exercise<br>C : exercise therapy solely	Ultrasound-guided dry needling	Knee osteoarthritis	Patellofemoral and medial tibial compartments	VAS,	A achieved significant improvement compared to B and C<br>A vs. B: MD = -15.61, 95% CI [-25.49, -5.51], (p = 0.001)<br>A vs. C: MD = -19.90, 95% CI [-29.71, -10.08], (p < 0.001).
								KOOS-pain,	A achieved significant improvement compared to B and C (A vs. B: MD = 9.76, 95% CI [2.38, 17.14], p = 0.006; A vs. C: MD = 9.48, 95% CI [2.31, 16.66], p = 0.010).

								KOOS-symptoms, KOOS-quality-of-life (QoL)	not statistically significant between groups.
Jin HP (2022) [30]	RCT	n=120(40/40/40)	A : Ultrasound-guided EA (Electro-acupuncture) at suprhyoid muscle	B: EA at CV23, GB12, GB20, etc. C : suprahyoid muscle according to anatomical location	Ultrasound-guided electroacupuncture	Pharyngeal dysphagia after stroke	Suprahyoid muscle	PAS score	PAS score: A < B, C ($p < 0.05$)
								IF scores	A > B, C ($p<0.05$).
								forward and upward movement distance of hyoid bone and thyroid carthilage	A was longer than that in B and the C ($p < 0.05$).
								incidence of subcutaneous hematoma	A 0% (0/40), < B 20.0% (8/40) , C 47.5% (19/40) ($p < 0.05$).

Xu H (2022)<br>[48]	RCT	n=63(33/30)	A : Ultrasound-guided hydrodilatation of glenohumeral joint combined with acupotomy	B : Only treated with ultrasound-guided hydrodilatation of glenohumeral joint)	Ultrasound-guided hydrodilatation of glenohumeral joint combined with acupotomy	frozen shoulder	Glenohumeral joint	Active ROM	AROM, CMS scores: A > B (all p< 0.05)
								CMS score	
								CHL thickness	CHL thickness, Rate of hypoechoic thickening in rotator cuff space: A < B (all p< 0.05)
								Rate of hypoecoic thickening in rotator cuff space	
Zheng Y (2014)<br>[53]	RCT	n=169(NA/NA)	A : UG-MSN	B : UG-DN	Ultrasound-guided Acupotomy, dry-needling	Chronic Neck pain	TrP	VAS, PCS, MCS	VAS: A < B (both p < 0.0001).<br>A also showed significantly lower scores on the adjusted neck disability index, and PCS
Krasny C (2005)<br>[32]	RCT	n=80(40/40)	A : Needling (US guided) + high-energy shockwave therapy	B : High-energy shockwave therapy	Ultrasound-guided dry needling	Calcific tendonitnis	Calcified deposit	Pain	A : 6.7 ± 2.6 → 13.3 ± 3.7 (p < 0.001)<br>B : 5.6 ± 2.3 → 10.6 ± 4.1 (p < 0.001)
								Daily activity	A : 11.0 ± 3.3 → 18.1 ± 4.2 (p < 0.001)<br>B : 10.7 ± 3.0 → 16.0 ± 3.9 (p < 0.001)
								Movement	A : 19.1 ± 6.4 → 32.7 ± 9.8 (p < 0.001)<br>B : 19.2 ± 6.4 → 29.7 ± 10.1 (p < 0.001)
								Power	A : 9.5 ± 4.4 → 12.7 ± 4.2 (p < 0.001)<br>B : 8.7 ± 3.5 → 11.0 ± 5.2 (p < 0.001)
Pan M (2019)	RCT	n=41(20/21)	A : Needle-knife (US guieded)	B : Needle-knife (blind release)	Ultrasound-guided acupotomy	Trigger finger	A1 pulley	clinical grade	A: Grade 0: 0 → 20 / Grade 1: 0 → 0 / Grade 2: 2 → 0/ Grade 3: 10 → 0/ Grade 4: 8 → 0<br>B: Grade 0: 0 → 4 / Grade 1: 0 → 15 /

[39]									Grade 2: 2→0 / Grade 3: 8→1 / Grade 4: 11→1
								complications	No any complications had been happened in the A group.
								operation time	A: 15.21 ± 0.87min<br>B: 5.23 ± 0.55min<br>A > B (p < 0.05)
Zhou Q (2023) [40]	RCT	n=100(NA/NA)	A :Acupotomy (US guided)	B : Acupotomy (non US guided)	Ultrasound-guided acupotomy	Anatomical study	Carpal ligament	Injury rate	A 0% vs B 6%, 12%, 20% (the rate of nerve, blood vessel and tendon damage) (P < 0.05)
								width of the transverse carpal ligament	A 86% vs B 36% (PL < 0.05)
Qiu Z (2022) [41]	RCT	n=84(28/28/28)	A : ultrasound-guided needle knife pushing group	B : non-ultrasound-guided needle knife pushing group<br><br>C : classical needle knife operation puncture group	Ultrasound-guided needle knife	A1 pulley release	A1 pulley	Relevant anatomical data	Injured cases: A 29 (20.7%) / B 36 (25.7%) / C 28 (20.0%)
									Missed release cases: A 8 (5.7%) / B 4 (2.9%) / T 13 (9.3%)
									Percentage of released A1 pulley: A 71.4% ± 30.7% / B 66.0% ± 20.3% / C 61.0% ± 30.4%
									Full release rates of the groups: A (31.4%) > B (15.7%) > C (13.6%)

Lin S (2024) [54]	RCT	n=100(50/50)	A : Regular acupuncture (US-guided)	B : shallow acupuncture	Ultrasound-guided acupuncture	Chronic subjective dizziness (CSD)	Baihui, Yintang, Taiyang, Tinggong, Wangu, Fengchi, Hegu, Fenglong, Taichong	Clinical effectiveness	A (94%) > B (80%) (p = 0.037)
								PSQI	A: 15.37 ± 7.82 → 6.83±3.65<br>B: 15.98 ± 10.83 → 8.18 ± 4.05<br>A < B (p < 0.05)
								DHI	A: 51.37 ± 16.89 → 32.73 ± 5.41<br>B: 50.65 ± 15.81 → 37.81 ± 7.52<br>A < B (p < 0.05)
								HAMD	A : 15.36 ± 7.18 → 5.87 ± 3.26<br>B : 15.51 ± 7.82 → 7.84 ± 3.98<br>A < B (p < 0.05)
								fatigue Severity Scale (FSS)	A : 33.48 ± 13.78 → 14.96 ± 6.98<br>B : 33.92 ± 14.05 → 18.23 ± 8.62<br>A < B (p < 0.05)
								HAMA	A : 21.65 ± 11.72 → 7.96 ± 4.81<br>B : 21.23 ± 11.54 → 11.78 ± 5.98<br>A < B (p <0.05)
Wang (2023) [55]	RCT	n=106(53/53)	A : musculoskeletal ultrasound guided acupuncture	B : conventional ultrasound-guided acupuncture	Ultrasound-guided acupotomy	osteoarthritis	nodules and cords in knee	VAS	A (4.3±0.7) < B (p < 0.05)
								Lysholm scale	pain, restlessness, atresia, swelling, limping, stair climbing, and squatting scores : A < B (p < 0.05)<br>support scores : not dramatically different between two groups (p > 0.05)
Dai J (2023) [52]	RCT	n=74(37/37)	A : deep acupuncture group (US-guided)	B : shallow acupuncture group (US-guided)	Ultrasound-guided acupuncture	N/A (bladder in controlling urine)	Zhibian (BL54)	PSV	A : 39.96 → 52.55<br>B : 41.50 → 47.55<br>A >B (p < 0.05)
								TAMX	A : 8.63 → 12.54<br>B : 10.13 → 10.95<br>A > B (p < 0.05)

								EDV	A : 2.26 → 2.34<br>B : 1.56 → 1.63<br>A > B (p < 0.05)
								PI	A : 5.62 → 4.99<br>B : 4.59 → 4.57<br>A > B (p < 0.05)
								RI	A : 0.95 → 0.95<br>B : 0.97 → 0.95<br>A ≒ B (p < 0.05)
								Bladder Volume	A : 25.27 → 50.70<br>B : 30.56 → 40.48<br>A > B (p < 0.05)
								C-MASS	A : 42.30, B : 9.03<br>A > B (p<0.01)
Guner D (2023)<br>[31]	RCT	n=44(22/22)	A : Ultrasound-Guided Dry Needling group	B : Physical exercise treatment group	Ultrasound-Guided Dry Needling	Piriformis muscle syndrome (PMS)	piriformis muscle	VAS	A : 7.6 ± 1.6 → 2.5 ± 2.1<br>B : 7.8 ± 0.7 → 2.6 ± 1.1<br>A ≒ B (p > 0.05)
								ODI	A : 20.9 ± 8.5 → 7.4 ± 5.7<br>B : 32.6 ± 6.7 → 9.9 ± 7.7<br>A ≒ B (p > 0.05)
								LEFS	A : 41.7 ± 15.9 → 69.4 ± 12.9<br>B : 42.4 ± 15.8 → 71.3 ± 8.2<br>A ≒ B (p > 0.05)
								DN4	A : 3.5 ± 2.1 → 0.9 ± 1.2<br>B : 4.1 ± 2.6 → 1.3 ± 1.4<br>A ≒ B (p > 0.05)
Zhu (2024)<br>[42]	RCT	n=70(35/35)	A : Ultrasound-Guided group	B : Without Ultrasound-Guided group	Ultrasound-guided acupuncture	lumbar disc herniation	Jiaji (EX-B2)	VAS	A : 5.49 ± 1.01 → 0.57 ± 0.61<br>B : 5.23 ± 1.03 → 1.86 ±1.03<br>A < B (p < 0.01)
								ODI)	A : 43.91 ± 10.02 → 5.71 ± 8.40<br>B : 41.17 ± 13.00 → 24.86 ± 14.35<br>A < B (p<0.01)
								JOA	A : 15.43 ± 2.21 → 25.37 ± 2.95<br>B : 16.74 ± 2.65 → 22.86 ± 2.52

									A > B (p < 0.01)
								MOS SF-36	A : 75.54 ± 8.22 → 83.97 ± 11.79<br>B : 71.83 ± 8.07 → 79.31 ± 12.12<br>A > B (p > 0.05)
Pu J (2023)<br>[57]	RCT	n=160(80/80)	A : ultrasound-guided injection acupotomy	B : ultrasound-guided selective nerve root block (SNRB)	Ultrasound-guided Acupotomy	cervical spondylotic radiculopathy (CSR)	A : posterior tubercle of the cervical vertebral transverse process, cervical nerve root<br>B : cervical nerve root	Odom's criteria clinical curative effect	A : 93.6% vs B : 81.0%<br>A > B (p = 0.018)
								VAS	A : 6.1 → 1.0<br>B : 6.3 → 1.8<br>A < B (p = 0.03)
								NDI	A : 51.4 ± 13.3 → 15.4 ± 12.8<br>B : 51.8 ± 13.0 → 21.9 ± 16.2<br>A > B (p = 0.006)
								SF-36	A : 43.3 ± 17.5 → 80.1 ± 12.6<br>B : 44.0 ± 16.5 → 72.6 ± 19.1<br>A > B (p = 0.004)
Lin Q (2005)<br>[58]	nRCT	NA	A : treatment group (electro-acupuncture with strong stimulation)	B : medication<br>C : conventional acupuncture	Dry needling	Upper segment ureterolithiasis	NA	Cure rate	A > B, C (p < 0.05)
								Total effective rate	A > B, C (p < 0.01)
Arias-Buría JL (2023)<br>[59]	nRCT	n=100(50/50)	A : Ultrasound-guided group	B : Palpation-guided group	Ultrasound-guided needling	Anatomical study	Interface between the patellar tendon and Hoffa's fat pad	Distance to the targeted	A : 0.25 ± 0.65mm<br>B : 2.5 ± 1.9 mm<br>A < B (p < 0.001)
								Time of the procedure	A : 54.8 ± 26.8 sec<br>B : 23.75 ± 15.4 sec<br>A > B (p < 0.001)
								Accuraterate of insertions	A : 100%<br>B : 80%<br>A > B

								Tissue number of passes	A : 2.55 ± 1.9<br>B : 1.5 ± 0.95<br>A > B (p = 0.001)
								Unintentional puncture structures	A : 16%<br>B : 52%<br>A < B (p < 0.001)
Malo-Urriés M (2024)<br><br>[60]	nRCT	n=100(50/50)	A : Ultrasound-guided group	B : Palpation-guided group	Ultrasound-guided needling	Anatomical study	Interface between the plantar fascia and flexor digitorum brevis	Distance to interface	A : 0.2 ± 0.7<br>B : 3.5 ± 2.2<br>A < B (p < 0.001)
								Longitudinal contact of the needle	A : 5.3 ± 2.2<br>B : 0.6 ± 1.8<br>A > B (p < 0.001)
								Time required	A : 53.8 ± 18.9<br>B : 19.1 ± 6.5<br>A > B (p<0.001)
								Tissue number of passes	A : 2.8 ± 1.5<br>B : 1.7± 0.9<br>A > B (p < 0.001)
								Unintentional puncture structures	A : 5 (10%)<br>B : 9 (18%)<br>A < B (p=0.249)

Notes. NA: not available; VAS: visual analog scale; HSS: hospital special surgery index; M-JOA: modified lumbago assessment by japanese orthopedic association; AOFAS-AH: american orthopedic foot and ankle society ankle-hindfoot scoring system; BMI: body mass index; SNK: small-needle-knife; OS: open surgery; QG: Quinnell grading; ESWT: extracorporeal shock wave; USGAP: ultrasound-guided acupotomy; NRS: numeral rating scale; SPADI: shouler pain and disability index; US: ultrasound; DN: dry-needling; TrP: trigger point; MTrP: myofascial trigger point; ADL: activities of daily living; PRI: pain rating index; PPI: present pain index; NDI: neck disability index; CPT: conventional physical therapy; VISA-P: Victoria institute of sports assessment-patellar tendinopathy; KOOS: knee injury and osteoarthritis outcome score; PPT: pressure pain threshold; ROM: range of motion; JOA: japanese orthopedic association; ODI: Oswestry disability index; LBOS: low back pain outcome scale; MSN: miniscalpel-needle; BCTQ: Boston carpal tunnel questionnaire; SSS: symptom severity scale; FSS: functional status scale; CSA: cross-sectional area; DML: distal motor latency; CMAP: compound muscle action potential; SNAP: sensory nerve action potential; SNCV: sensory nerve conduction velocity; PRTEE: patient rated tennis elbow evaluation; NPRS: numeric pain rating scale; MCS: mental composite score; PCS: physical composite score; SF-36: short-form 36 health survey; MPQ: McGill pain questionnaire; PAS: penetration aspiration scale; IF: Ichiro Fujima ingestion swallowing functions score; CMS: Constant-Murley scale; CHL: coracohumeral ligament; UG-MSN: ultrasound-Guided Miniscalpel-Needle Release; UG-DN: ultrasound-guided dry needling; PSQI: Pittsburgh Sleep volume; DHI: Dizziness Handicap Inventory; HAMD: Hamilton Depression Scale; HAMA: Hamilton Anxiety Scale; PSV: Peak systolic velocity; TAMX: Time average maximum velocity; EDV: End diastolic velocity; PI: Pulsatility index; RI: Resistance index; LEFS: Lower Extremity Functional Scale; DN4: Douleur Neuropathique 4 questionnare score;
